# Luminescence Thermometry Beyond the Biological Realm

**DOI:** 10.1021/acsnanoscienceau.3c00051

**Published:** 2023-12-01

**Authors:** Benjamin Harrington, Ziyang Ye, Laura Signor, Andrea D. Pickel

**Affiliations:** † Materials Science Program, University of Rochester, Rochester, New York 14627, United States; ‡ The Institute of Optics, University of Rochester, Rochester, New York 14627, United States; § Department of Mechanical Engineering and Materials Science Program, University of Rochester, Rochester, New York 14627, United States

**Keywords:** luminescence thermometry, nanothermometry, thermal metrology, upconverting nanoparticles, color centers, ratiometric thermometry, measurement artifacts, batch calibration

## Abstract

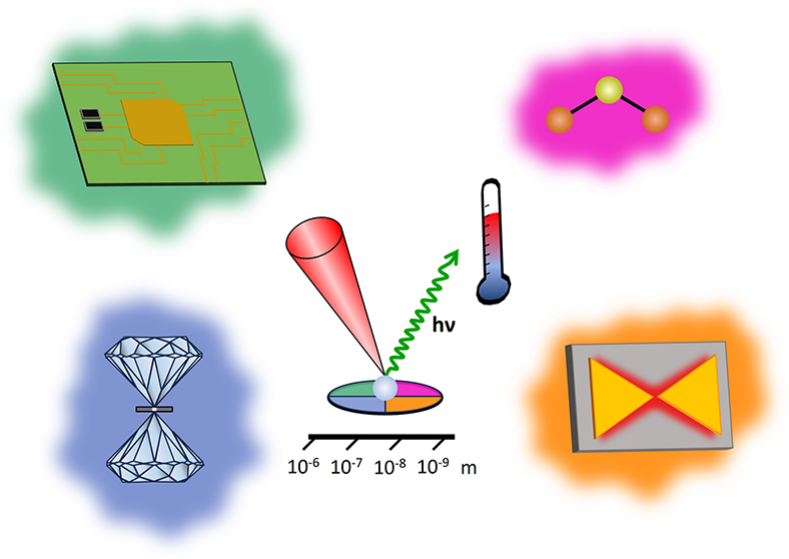

As the field of luminescence thermometry has matured, practical applications of luminescence thermometry techniques have grown in both frequency and scope. Due to the biocompatibility of most luminescent thermometers, many of these applications fall within the realm of biology. However, luminescence thermometry is increasingly employed beyond the biological realm, with expanding applications in areas such as thermal characterization of microelectronics, catalysis, and plasmonics. Here, we review the motivations, methodologies, and advances linked to nonbiological applications of luminescence thermometry. We begin with a brief overview of luminescence thermometry probes and techniques, focusing on those most commonly used for nonbiological applications. We then address measurement capabilities that are particularly relevant for these applications and provide a detailed survey of results across various application categories. Throughout the review, we highlight measurement challenges and requirements that are distinct from those of biological applications. Finally, we discuss emerging areas and future directions that present opportunities for continued research.

## 1 Introduction

Temperature is a fundamental parameter across areas ranging from device reliability to catalytic efficiency to life-sustaining cellular processes, and with this broad relevance comes an inherent need for effective and reliable temperature measurements. As a result, a vast library of thermometry techniques has been developed, with options spanning a wide range of costs, compatibility with different operating environments, and spatial, temperature, and temporal resolution. Common thermometry techniques employed across consumer, industrial, and research settings include electrical sensors such as resistance temperature detectors, thermistors, and thermocouples, scanning probe methods like scanning thermal microscopy (SThM), and optical techniques including thermoreflectance, Raman, thermal radiation, and luminescence-based approaches. The continued downscaling of modern devices has led to an ongoing emphasis on temperature probes with micro to nanoscale spatial resolution.^1^ A major advantage of optical thermometry techniques is their noninvasive or less invasive nature,^2^ stemming from the fact that these methods use lasers or other illumination sources to remotely probe the sample or, in the case of thermal radiation-based approaches, rely on the thermal emission from the sample itself. Luminescence thermometry falls within this category of optical techniques, which are often referred to as “non-contact” methods, although luminescence thermometry has also been referred to as a “semi-contact”^3^ or “remote detection”^4^ method since the probe is placed directly on the sample while its emission is collected from the far field. Similar situations exist for some other optical thermometry techniques like thermoreflectance methods that require depositing a metal transducer layer on the sample surface^5,6^ and Raman measurements on surfaces using molecular probes.^7^ Meanwhile, thermal radiation, certain Raman approaches based on solid materials,^8^ and emerging transducer-less thermoreflectance methods^9−11^ can be considered truly noncontact.

**Figure 1:**
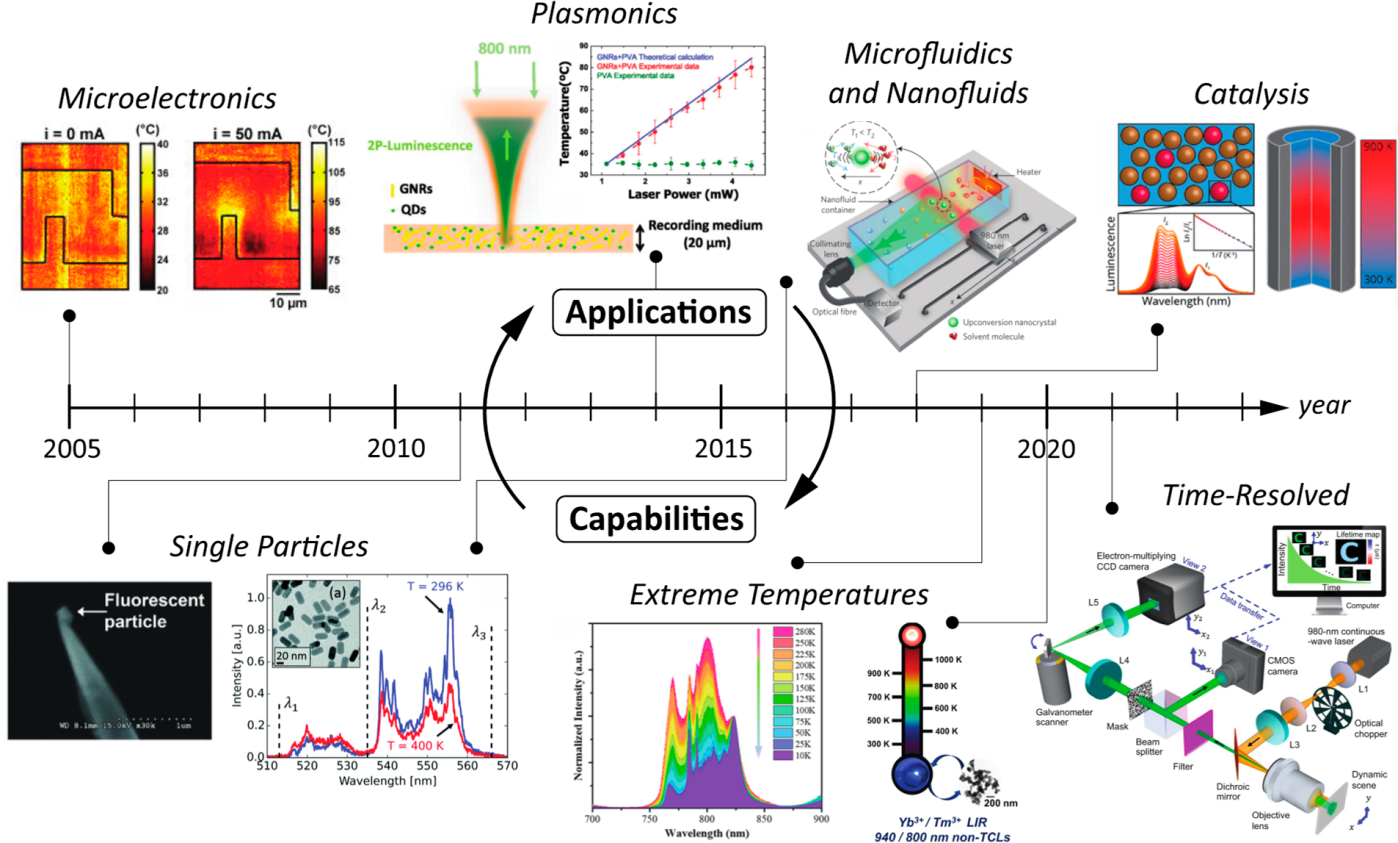
While luminescence thermometry has both longstanding ties to and continued promise for biological applications, a wide variety of nonbiological applications have emerged over the last several decades. Many of these applications require pushing the bounds of existing techniques to achieve broader operating temperature ranges or higher spatial and temporal resolution. In turn, these advanced capabilities drive the growth of new applications. Top row images, left to right: Reprinted from ref (12) with the permission of AIP Publishing. Reprinted from ref (13) with the permission of AIP Publishing. Reprinted with permission from ref (14) Copyright 2016, Springer Nature Limited. Reprinted with permission from ref (15) Copyright 2018 American Chemical Society. Bottom row images, left to right: Reprinted with permission from ref (16) Copyright 2011 WILEY-VCH Verlag GmbH & Co. KGaA, Weinheim. Reproduced from ref (17) with permission from the Royal Society of Chemistry. Reprinted with permission from ref (18) Copyright 2019 American Chemical Society. Reprinted with permission from ref (19) Copyright 2020 American Chemical Society. Reprinted with permission under a Creative Commons CC BY 4.0 license from ref (20) Copyright 2021 Springer Nature.

Luminescence thermometry is deeply intertwined with biological imaging due to the fact that many luminescent thermometers were first developed as biological imaging probes. These probes have a variety of features that are particularly desirable for in vivo applications: many are excited or emit within the first and second biological windows, near-infrared (NIR) wavelength regions where absorption and scattering by biological tissue is minimized, and their surfaces are amenable to modifications that allow for diverse bioconjugation strategies. Furthermore, many luminescent thermometers possess the small probe size, high spatial resolution, and sub-1 K temperature resolution desired for probing temperature in cellular environments. While the advantages of luminescence thermometry for biological applications are clear, one might ask why luminescence thermometry would be selected over other optical techniques for nonbiological applications. In addition to the desirable properties that it shares with other optical thermometry methods, such as remote detection and submicron spatial resolution, luminescence thermometry has other features that can facilitate unique measurement capabilities and applications (Figure 1). Luminescent probes are thermally stable and can operate over exceptionally wide temperature ranges, from cryogenic temperatures up to ∼1000 K. Many luminescent probes are chemically inert and can operate during chemical reactions without perturbing the reaction. Luminescent thermometers have a broad and tunable range of excitation and emission wavelengths, providing excellent compatibility with applications where certain spectral regions must be avoided and enabling optical multiplexing. The nanoscale size and discrete nature of luminescent probes also offer unmatched flexibility in terms of how the probes can be distributed on the sample of interest, from dense layers or ensembles to single-particle measurements.

This review complements several other recent reviews on luminescence thermometry, some of which specifically focus on biological applications^21−24^ and some of which also cover a subset of nonbiological applications.^2−4,25−27^ While a number of existing reviews provide excellent summaries of the different luminescent probes and signals that can be exploited for thermometry, here we largely focus on the applications enabled by these techniques. We also omit coverage of thermographic phosphors used primarily for combustion and industrial manufacturing applications,^28^ which are typically applied as paints or thin film coatings and have been reviewed elsewhere,^29,30^ and focus on luminescent nanomaterial-based thermometry. The review is organized as follows: we first provide an overview of upconverting nanoparticles (UCNPs) and related lanthanide-containing materials, nitrogen vacancy (NV) and other color centers, quantum dots (QDs), and other luminescent probes. These particular luminescent probes are the most commonly used materials across the literature we cover in the later section focused on applications of luminescence thermometry. Subsequently, we discuss several specific capabilities, including high-temperature and cryogenic thermometry, single-particle measurements, and time-resolved thermometry. We then present applications of luminescence thermometry to microelectronics and other devices, plasmonics, catalysis, optical trapping and levitation, pressure sensing, microfluidics, and additional selected applications. Finally, we discuss emerging areas such as the identification and mitigation of artifacts that can distort luminescence thermometry signals and the resulting temperature measurements, the application of advanced data analysis and machine learning (ML) methods to luminescence thermometry, and particle-to-particle uniformity of luminescence thermometry signals and the use of batch calibration procedures. Several of the emerging areas we cover were also highlighted in a very recent comprehensive review,^4^ underscoring the growing importance of these topics within the luminescence thermometry community. We end by summarizing the current status of the field and offer a perspective on future research directions. Throughout the review, we emphasize motivations and applications that largely fall within the physical sciences and nonbiological engineering disciplines. Nonetheless, given the strong ties between luminescence thermometry and biology and the fact that certain requirements are shared between biological and nonbiological applications, we inevitably also touch on work that is primarily biologically motivated.

## 2 Probes

### 2.1 Upconverting Nanoparticles (UCNPs) and Related Lanthanide-Containing Materials

UCNPs are rare earth-doped luminescent probes with diverse applications including biological imaging, theranostics, anticounterfeiting, lasing, and sensing of quantities including pH, viscosity, and temperature. UCNPs commonly consist of multiple rare earth dopants in fluoride, oxide, or other crystalline host matrices. Often, one lanthanide dopant serves as the sensitizer, which absorbs the excitation light and subsequently transfers energy to another dopant that serves as the activator, which then emits luminescence. The most common doping composition is 20% Yb^3+^ and 2% Er^3+^, but various other rare earth dopants including Nd^3+^,^31−34^ Tm^3+^,^35−39^ Ho^3+^,^40,41^ Eu^3+^,^42,43^ Gd^3+^,^31,44,45^ and Pr^3+^^45−48^ are also routinely employed. The most common host matrix material is hexagonal (β) phase NaYF_4_, which has a low maximum phonon energy that minimizes nonradiative recombination. A range of other host matrix materials have also been explored. Other UCNP configurations beyond the sensitizer-activator model, such as an Er^3+^ sublattice that promotes thermally activated upconversion luminescence,^49^ can also enable thermometry with very high temperature sensitivity. UCNPs traditionally absorb two or more NIR photons and emit a single photon in the visible or ultraviolet wavelength range. UCNPs that convert longer wavelength NIR excitation light to shorter wavelength NIR emission have also been developed. UCNPs are favorable for biological imaging and sensing due to their excitation and in some cases emission wavelengths falling within the biological transparency windows, lack of autofluorescence, and increased optical penetration depth in tissue relative to other common probes.^50^ UCNPs also have desirable features for nonbiological applications such as wide operating temperature ranges, excellent thermal stability, and chemical inertness. Other features such as UCNPs’ lack of photobleaching or blinking and the broad tunability of their excitation and emission wavelengths are beneficial across all application categories. Related lanthanide-containing materials, such as lanthanide metal–organic frameworks (MOFs),^51^ also facilitate thermometry based on similar operating principles.

Many lanthanides have closely spaced, thermally coupled energy levels whose relative population is governed by Boltzmann statistics. As a result, the relative emission intensity corresponding to the transitions from each excited state to the ground state provides a convenient temperature-dependent metric.^52^ The emission wavelength range is typically divided into two bands, again corresponding to the transitions from each excited state to the ground state, and a luminescence intensity ratio *r* is defined as the integrated emission intensity from the higher energy, shorter wavelength band divided by that from the lower energy, longer wavelength band. *r* increases with temperature and can be calibrated to an Arrhenius-type relation of the form , where *A* is a constant related to the radiative transition rates from each excited state to the ground state, Δ*E* is the energy gap between the two excited states, *k*_B_ is the Boltzmann constant, and *T* is temperature. This approach, known as “ratiometric” thermometry, is the most common UCNP thermometry method. Ratiometric sensing is advantageous over approaches based on absolute intensities since ratiometric signals are often insensitive to intensity variations resulting from sample absorption and scattering and day-to-day alignment variations, for example, although recent work discussed further in Section 5.1 (Measurement Artifacts) has demonstrated that parameters including the excitation laser intensity and surrounding environment can affect UCNP ratiometric thermometry signals under certain circumstances and caution must be taken.^53−55^ Ratiometric thermometry traditionally requires calibration using a temperature-controlled platform combined with a reference temperature probe, meaning that these approaches are by definition secondary thermometry methods. However, strategies for applying UCNPs for primary thermometry, in which temperature can be determined directly from a known physical law with no required calibration, have also been reported.^56^

Figures of merit used to characterize the performance of luminescent thermometers include the absolute and relative thermal sensitivities, operating temperature range, repeatability, and spatial, temporal, and temperature resolution. Other reviews have provided extensive summaries of different UCNP compositions that have been used for thermometry and comparisons of the relevant figures of merit.^57,58^ Similar summaries and comparisons of these figures of merit for other luminescent materials discussed in subsequent sections are also available in other review articles.^2,21,25^ Here, we summarize a selection of recent, representative work on UCNP thermometry. The ^2^H_11/2_ to ^4^I_15/2_ and ^4^S_3/2_ to ^4^I_15/2_ transitions of Er^3+^ is the most common set used for ratiometric thermometry,^59^ but numerous other dopants and transitions have also been employed to achieve higher sensitivities,^36,39,40,47,48,60^ broader operating temperature ranges,^33,41,45,46^ and wide-ranging excitation and emission wavelengths. For example, Nd^3+^ sensitization^31−34^ allows for excitation at 800 or 808 nm, where the absorption cross-section of water is much smaller than that at the 976 or 980 nm excitation wavelengths used for Yb^3+^-sensitized UCNPs. While UCNP-based ratiometric thermometry has most commonly relied on thermally coupled energy levels (TCLs), more recent work has also explored the use of nonthermally coupled levels (non-TCLs),^61^ which can provide higher sensitivities or extended operating temperature ranges. Stark sublevels of a single band have also been applied for ratiometric thermometry.^34,37^ Other strategies that have been used to increase temperature sensitivity include applying inert coatings to the UCNPs,^62^ engineering the phonon energies of the host matrix,^63^ and manipulating the temperature-dependent electron population through the use of an additional excitation laser.^64^ An emerging alternative strategy is to increase sensitivity by defining new temperature-dependent metrics, such as the product of the luminescence intensity ratio based on emission from two thermally coupled energy levels and that based on the emission from a single band upon successive excitation of two TCLs^42^ or the product of multiple luminescence intensity ratios from different pairs of thermally coupled energy levels.^65^ Recent work has also demonstrated how hardware considerations such as sensor noise can ultimately limit the temperature measurement precision.^66,67^ Multiparametric sensing approaches that combine several temperature-dependent parameters can also be used to improve sensitivity and are discussed in further detail in Section 5.2 (Advanced Data Analysis and Machine Learning Approaches).

Beyond ratiometric signals, other temperature-dependent UCNP emission signatures such as spectral peak shifts^68^ and luminescence lifetimes have also been used for thermometry. Several UCNP-based demonstrations of luminescence lifetime thermometry and other measurement schemes that enable real-time temperature measurements are discussed further in Section 3.4 (Time-Resolved Measurements).

### 2.2 Nitrogen Vacancy (NV) and Other Color Centers

NV centers are bright, photostable luminescent defects consisting of a substitutional nitrogen atom and an adjacent vacancy that are found in both bulk diamond and nanodiamonds. NV centers display magnetic sensitivity and long spin coherence times that are appealing for applications like magnetometry, quantum information processing, and quantum sensing. Due to these features, NV centers are more commonly employed for nonbiological applications than other luminescent probes; nonetheless, other features including their temperature sensitivity and amenability to surface functionalization are also attractive for biological applications such as nanoscale thermometry in a living cell.^69^ Recent reviews dedicated exclusively to diamond thermometry provide detailed coverage of NV center thermometry fundamentals as well as biological and other applications.^70,71^ The most common NV center thermometry approach is optically detected magnetic resonance (ODMR) thermometry, where the NV center spin state is manipulated using microwave pulses and the temperature-dependent zero-field splitting of the electronic ground state spin sublevels can be determined from the emitted luminescence.^72^ The temperature dependence of the zero-field splitting has been attributed to a combination of local thermal expansion and electron–phonon interactions.^73^ Very recent work has put forth a physically motivated analytical expression where the zero-field splitting shifts in proportion to the occupation of two representative phonon modes, which matches experimental measurements from 15 to 500 K.^74^

NV center thermometry with noise floors at or below 10 mK/Hz^1/2^ and temperature resolution down to the mK level has been achieved,^75^ in some cases using enhancement strategies such as those based on magnetic criticality^76^ and spin coherence.^77,78^ NV center thermometry is robust over an exceptionally wide temperature range, from cryogenic temperatures up to 600 K,^79^ and both NV centers in bulk diamond and NV center-containing nanodiamonds are chemically inert and thermally stable at elevated temperatures. Single NV centers in high-quality bulk diamond typically achieve the highest temperature sensitivities. Meanwhile, nanodiamonds containing either single or multiple NV centers are advantageous in terms of their potential to reduce parasitic heat sinking effects, particularly given the high thermal conductivity of bulk diamond, and enable single-particle temperature measurements. ODMR thermometry has also been combined with other sensing modalities for multiplexing applications such as simultaneous magnetometry and thermometry.^80,81^ Very recently, a hand-held device enabling ODMR thermometry was demonstrated, suggesting a path toward practical deployment of ODMR thermometry at macroscopic length scales in industrial or medical settings.^82^

Because ODMR thermometry requires the use of a microwave antenna, which increases instrumentation complexity and can cause parasitic heating or otherwise disturb sensitive samples, alternative all-optical thermometry approaches have also been developed. Negatively charged NV centers display a zero-phonon line (ZPL) at approximately 638 nm, corresponding to the purely electronic transition. The ratio of the area under the ZPL relative to the total area under the emission band is known as the optical Debye–Waller factor and has been used for all-optical NV center thermometry,^83^ along with a related approach based on the amplitude of the background emission at the ZPL center wavelength relative to the ZPL amplitude.^84^ A limited number of studies have also explored the use of NV center excited state lifetimes for thermometry,^79,85,86^ providing another all-optical approach, which is discussed further in Section 3.4 (Time-Resolved Measurements). More recently, other color centers beyond NV centers have gained traction for thermometry, such as silicon vacancy (SiV),^87−91^ germanium vacancy (GeV),^92−94^ tin vacancy (SnV),^95^ and magnesium vacancy (MgV)^94^ centers, all of which enable all-optical thermometry. A diverse range of physical mechanisms gives rise to the temperature-dependent emission signals used for color center-based thermometry. Temperature-dependent zero-field splitting and changes to the ZPL peak position or width are common metrics for these other color centers in addition to NV centers. Other signals, such as anti-Stokes to Stokes photoluminescence intensity ratios whose temperature dependence originates from thermally activated anti-Stokes excitation^93^ or the emission intensity resulting from thermally activated two-photon excitation,^91^ have also been employed.

### 2.3 Quantum Dots (QDs)

QDs are a class of nanoparticles with important applications across display technologies, solar cells, bioimaging, and in the sensing of pH, voltage, and temperature.^96−99^ Semiconductor-based QDs, particularly group II-VI QDs such as CdSe,^13,100−103^ ZnS,^13,97,98,102^ and CdTe,^100,104^ are some of the most common types. QDs are typically spherical in nature, although related particles known as quantum rods, with an elongated length of up to ∼100 nm, have also been synthesized.^105^ Alternatives such as carbon-based QDs^106−108^ and group I-III-VI_2_ compounds have been developed due to concerns over both the cellular and environmental toxicity of heavy metal QD materials.^106^ Many groups have also developed multilayer QDs consisting of varying compositions of cores and multiple layers of shells^99,103,109−111^ and have incorporated QDs in composites with other types of nanoparticles.^112,113^ Typical QDs are smaller than 20 nm in size and include only a few hundred or thousands of atoms within the lattice,^99^ leading to quantum confinement effects since the characteristic size of the QD is comparable to the exciton Bohr radius.^99^ QDs have a discrete valence and conduction band, which gives rise to a forbidden energy bandgap in between. Bound electron–hole pairs (excitons) are created across the valence and conduction bands and later recombine. Radiative recombination produces the QD luminescent response with emission energy determined by the bandgap of the material. QDs offer a high quantum yield^106^ and good intensity stability compared to some other probes like organic dyes.^103^ QDs are most commonly utilized for thermometry at biologically relevant temperatures since thermal quenching occurs at higher temperatures due to an increased rate of nonradiative transitions,^99^ although work is being done to expand the operating temperature range of these probes.^100^

The temperature-dependent luminescence response of QDs is typically modeled with the Varshni equation,^96,100,105^ a widely used empirical expression that describes how the bandgap of a semiconductor changes with temperature. Modifications made to the physical structure of QDs including to the size, shape, and composition have also been shown to affect the bandgap and thus the temperature-dependent response.^96,99^ A detailed summary of QD structural and compositional modifications and the resulting photophysical changes relevant for thermometry is available for further review.^98^ On the one hand, the tunability of QDs enables access to a large range of possible emission wavelengths, but relatively unpredictable or unintended structural changes can introduce uncertainty in temperature measurements across experiments without proper calibration.^100^ QDs exhibit several different temperature-dependent signals including photoluminescence (PL) lifetimes, PL intensity,^111^ PL peak width, and PL peak wavelength.^99^ CdSe QDs are commonly used for luminescence nanothermometry based only on the observed wavelength shift of their PL emission peak.^13,101,102,105^ More broadly, however, QD PL intensity changes and peak wavelength shifts have been found to be sensitive to nonthermal effects, making temperature readings difficult with these approaches alone.^99^

Another thermometry approach for QDs utilizes the ratiometric intensity between the PL emission of a primary QD and a second emission signal^97,99^ originating from other dopants, QD layers, or nanoparticles for improved sensitivity.^96^ This “double emission” or “dual emission” technique is commonly achieved by doping the host QD with an additional emitter—typically Mn^2+^, Ag^+^, and Cu^+^ are used^98,99,114^—or by layering additional QDs onto the primary QD in a core/shell approach.^99,109,110^ Zhao et al.^99^ provided a comprehensive review of work done to synthesize novel core/shell and core/shell/shell QDs for ratiometric thermometry. In a unique demonstration of the double emission approach, a hybrid nanocrystal incorporating PbS QDs and NaYbF_4_:Tm^3+^ UCNPs utilized the ratiometric signal between the QDs and the UCNPs for nanothermometry.^113^ Because the emission overlapped in the spectral domain, time-resolved measurements were used to separate the signals from the two emitters based on differences in their luminescence lifetimes.

### 2.4 Other Probes

Beyond the three specific classes of probes described above, numerous other luminescent materials have also been applied for thermometry. Here, we cover a limited selection of other common luminescent thermometers that are relevant to work discussed in later sections. In recent years, transition metal (TM) ions such as chromium, manganese, vanadium, and titanium have also been applied for luminescence thermometry. TM-doped materials have much larger absorption cross-sections compared to those for lanthanide-doped materials, resulting in brighter luminescence intensity.^115^ Additionally, in contrast with lanthanide-doped materials whose temperature dependence is largely based on the selection of the rare earth elements, for TM-doped materials the host matrix and the resulting crystal field acting on the TM ions can dramatically affect the temperature-dependent emission.^116−118^ Generally, the temperature-dependent emission originates from the temperature dependence of both the radiative and nonradiative relaxation rates, in contrast with lanthanide-doped materials where the radiative relaxation is typically assumed to be temperature independent.^115^ The temperature-dependent radiative and nonradiative relaxation rates can be indirectly observed via changes in parameters such as PL intensities and lifetimes. We highlight Cr^3+^ as one of the most frequently investigated TM ions. Here, common approaches include ratiometric thermometry based on the relative emission intensities of the bands corresponding to the ^2^E to ^4^A_2_ and ^4^T_2_ to ^4^A_2_ transitions or thermometry based on the lifetimes of these excited states.^119−123^ The same transitions have also been applied for thermometry using Mn^3+^ or Mn^4+^, another popular TM ion.^124^

Another relevant class of compounds is organic thermometers such as fluorescent dyes, which are highly biocompatible and have longstanding applications for imaging, pH sensing, and thermometry in a biological context. Nonetheless, these same materials have also been used in nonbiological thermometry applications, some examples of which will be discussed in later sections. The temperature-dependent fluorescence emitted by organic molecules typically originates from the temperature dependence of nonradiative decay processes such as internal conversion or intersystem crossing.^125^ In most cases, the fluorescence spectrum consists of a single broad peak, and the temperature dependence can manifest as changes in the fluorescence intensity^126,127^ or lifetime.^128^ For fluorescent dyes, the temperature dependence can also be influenced by processes such as Förster resonance energy transfer^129,130^ or twisted intramolecular charge transfer.^131^ To circumvent the challenges associated with thermometry based on absolute fluorescence intensity, a common strategy is to use a ratiometric signal based on the emission intensities of two dyes with different temperature sensitivities.^132−134^ A similar approach has been used to create a ratiometric thermometer based on two fluorescent proteins with different temperature sensitivities.^135^ Ratiometric thermometry methods can also be developed for organic molecules by linking multiple emitting species via a MOF or a polymer matrix.^118,136−139^

## 3 Capabilities

### 3.1 High-Temperature Measurements

Several of the emitters discussed thus far have been evaluated for use specifically in high-temperature environments. High-temperature measurements are of growing importance for studying high-power devices^140^ and for investigating processes including metal alloy formation, sintering, and catalytic reactions.^19^ In the most extreme cases, relevant industrial applications for luminescence thermometry such as evaluating thermal barrier coatings for gas turbine blades can require operating temperatures as high as 1500 K.^141^ High-temperature measurements can also facilitate fundamental studies of material properties under extreme conditions. Many UCNPs suffer from catastrophic thermal quenching and host material degradation around 600 K,^142^ with noticeable sensitivity losses occurring at even lower temperatures.^143^ Several studies have improved upon the high-temperature capabilities of UCNPs and related upconverting materials to measure temperatures as high as 1000 K.^19,140,141^ Meanwhile, diamond has exceptional high-temperature stability, and temperature-dependent responses from NV centers including all-optical^86^ and ODMR^79^ signals remain robust up to approximately 700 K.

Geitenbeek et al.^141^ utilized bare NaYF_4_:Yb^3+^,Er^3+^ UCNPs for high-temperature thermometry up to 600 K; however, above these temperatures the particles melted and fused together. Incorporating a SiO_2_ shell around the UCNP core prevented this issue, thereby enabling thermometry up to 900 K. A similar study found that a SiO_2_ shell enabled higher temperature measurements with LiLuF_4_:Yb^3+^, Er^3+^ UCNPs up to 800 K.^140^ As an alternative to the shell encapsulation technique, a study involving LiYF_4_:Yb^3+^,Er^3+^ upconverting microcrystals used varying levels of Cu^2+^ doping to reinforce the crystalline host matrix, mitigate defects, and reduce thermal quenching of the luminescence response for thermometry up to 873 K.^142^ Recent work identified temperature-dependent cross-relaxation processes in Pr^3+^-doped Y_3_GaO_6_ as a mechanism for achieving ratiometric thermometry from room temperature up to approximately 800 K.^143^ This study also found that varying the Pr^3+^ concentration modified the thermal sensitivity across the same temperature range. Other work involving UCNPs used a NIR ratiometric thermometry signal based on the ^2^F_5/2_ to ^2^F_7/2_ transition of Yb^3+^ and the ^3^H_4_ to ^3^H_6_ transition of Tm^3+^, which, notably, are non-TCLs, in YVO_4_:Yb^3+^,Tm^3+^ UCNPs for thermometry up to 1000 K.^19^ The non-TCLs studied showed significantly better thermal sensitivity and temperature resolution compared to TCLs of Tm^3+^ at elevated temperatures, highlighting the possibility that underexamined luminescence signals lacking good resolution and sensitivity at room temperature could potentially be well-suited for high-temperature thermometry.^19,140^

### 3.2 Cryogenic Measurements

Temperatures below 120 K are generally considered cryogenic, where research pertaining to the study of superconductivity, aerospace materials, and quantum devices often occurs.^144−146^ Contact-based thermometry measurements for cryogenic systems can pose particular challenges due to the often-unavoidable heat transfer between the relatively hot probe and a cold sample. As a result, a variety of luminescent thermometers have been developed specifically for cryogenic thermometry. Fukami et al.^147^ utilized the ZPL amplitude ratio of NV centers in nanodiamonds at temperatures ranging from 85 to 300 K. Across the cryogenic range, an important cutoff exists at 77 K, where liquid nitrogen cooling systems reach their lower temperature limit. Below this limit, helium-cooled systems are necessary as demonstrated by Chen et al.,^144^ who used the temperature-dependent line width of GeV centers in nanodiamonds for thermometry from 5 to 35 K. Additional work in the ultralow temperature range was performed by Zhao et al.,^148^ who demonstrated a Tb^3+^/Eu^3+^ MOF thermometer with tunable relative sensitivity for the 25 to 125 K range. A similar temperature range of 40 to 150 K was achieved using the ratiometric response from the thermally coupled ^4^T_2_ to ^4^A_2_ and ^2^E to ^4^A_2_ transitions of Cr^3+^ in a CaHfO_3_ perovskite host.^119^ Additional work has explored BiLaWO_6_:Yb^3+^,Er^3+^,Tm^3+^^149^ and BaGa_2_O_4_:Bi^3+^^150^ for thermometry from 30 to 300 K and 7 to 300 K, respectively.

Oftentimes, a particle composition with an existing thermometry signal near room temperature can incorporate additional emitters with a cryogenic temperature response, such that the modified composition is capable of measurements across both temperature ranges. This strategy was demonstrated for core/multishell UCNPs consisting of NaYbF_4_:Tm^3+^ cores and CaF_2_/NaYF_4_:Yb^3+^,Er^3+^/CaF_2_ shells.^18^ The intensity ratio originating from thermally coupled Tm^3+^ Stark sublevels was used as a temperature-dependent signal between 10 to 150 K since the conventional Er^3+^ emission was barely detectable below 150 K. By utilizing both the Tm^3+^ and Er^3+^ thermometry signals, a particle composition with broad applicability for thermometry from 10 to 295 K was demonstrated. Additional work with a Tb^3+^/Eu^3+^ phosphonate dimer^151^ and several different compositions of Tb^3+^/Eu^3+^ MOFs has established that these dopants can facilitate sensitive thermometry from approximately 10 K up to room temperature.^145,146,152^

In certain cases, a single emitter can provide thermometry capabilities across a wide range of temperatures. Brites et al.^46^ developed a Sr_2_GeO_4_:Pr^3+^ thermometer that is particularly notable for its broad detection capabilities at cryogenic temperatures, near room temperature, and even into the high temperature region, enabled by two distinct ratiometric signals operating from 17 to 300 K and 300 to 600 K. Bolek et al.^153^ likewise demonstrated thermometry between 17 and 700 K based on Y_3_(Al,Ga)_5_O_12_:Pr^3+^ phosphors using multiple ratiometric signals and the luminescence decay time. A broad detection range of 4 to 500 K was also achieved with an erbium-chloride-silicate nanowire using ratiometric signals derived from several distinct NIR Er^3+^ Stark sublevels.^154^

### 3.3 Single-Particle Measurements

**Figure 2:**
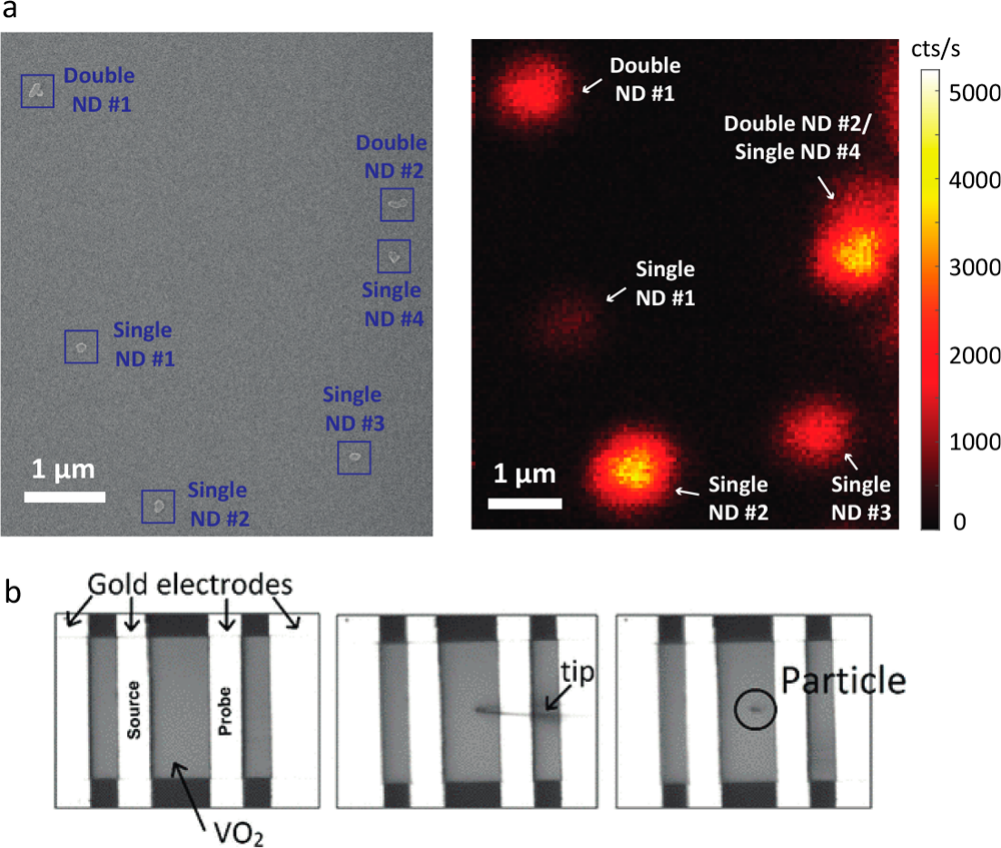
(a) SEM and fluorescence images of the same pattern of NV center-containing nanodiamonds on a substrate. Such cross-validation is particularly important for confirming the presence of isolated single particles when there is substantial particle-to-particle variation in emission intensity, as in this example. Reprinted from ref (85) with the permission of AIP Publishing. (b) Another approach to performing single-particle thermometry is to deterministically place a single particle at the location of interest using a nanomanipulator tip. Reprinted with permission from A. Zimmers et al., PRL **110**, 056601 (2013). Copyright 2013 American Physical Society.

In contrast with ensemble measurements that are fundamentally diffraction limited, single-particle thermometry can allow for temperature measurements with spatial resolution below the diffraction limit. When an isolated single particle is excited, the resulting temperature-dependent luminescence response corresponds solely to the location of that particle, and the spatial resolution of the temperature measurement is therefore determined by the particle size. Consequently, although single-particle measurements are technically demanding and challenges associated with particle-to-particle variation can play a larger role than in ensemble measurements,^155,156^ a number of the probes discussed thus far have been employed for single-particle thermometry. Key to performing single-particle measurements is the dispersal of individual particles and subsequent validation that the particles are in fact spatially isolated. Dispersal is often accomplished by spin coating a dilute solution of particles on a sample surface.^17,66,84,157^ Validation that a chosen particle is isolated (i.e., other particles are sufficiently far away such that only one particle falls within the excitation laser spot) is typically performed by observing the measurement region via scanning electron microscopy (SEM)^17,85,154^ (Figure 2a). Methods for deterministically placing a single-particle thermometer at the location of interest on a sample surface include nanomanipulation techniques such as nanomanipulation using an atomic force microscope (AFM) tip^85^ (Figure 2b). Lithographic approaches, including those based on lift-off processes^158^ and nanofluidic confinement,^159^ have successfully been used to position individual nanoparticles and thus offer another potential strategy for placing single-particle thermometers at desired locations. Optical trapping methods that can isolate and position single particles for thermometry have also been demonstrated and will be discussed later in Section 4.4 (Optical Trapping and Levitation). Single particles can also be attached to AFM tips and scanned over a sample.^12,16,160^ In contrast with single-point measurements using a particle at a fixed location on a sample surface, this approach allows for temperature mapping, although such measurements retain some of the challenges associated with SThM measurements such as unknown thermal resistances and parasitic heat sinking.

An early demonstration of thermometry via isolated single particles on a substrate was performed by Li et al.,^101^ who used the temperature-dependent spectral peak shifts of individual CdSe QDs nominally ∼7 to 12 nm in diameter. Zimmers et al.^161^ used an isolated, micron-wide Yb^3+^ and Er^3+^ codoped particle placed via nanomanipulation to study the insulator–metal transition of VO_2_. Others have similarly used individual Yb^3+^ and Er^3+^ codoped particles hundreds of nm to microns in size for thermometry.^66,142,157,162^ Kilbane et al.^17^ showed that the temperature-dependent response of individual NaYF_4_:Yb^3+^,Er^3+^ UCNPs was preserved for particles 20 × 20 × 40 nm^3^ in size, even though these small single particles require excitation intensities several orders of magnitude larger than those used for ensemble measurements. Pickel et al.^53^ later demonstrated similar results for individual 50 × 50 × 50 nm^3^ NaYF_4_:Yb^3+^,Er^3+^ UCNPs. Other morphologies have also been developed, such as elongated erbium-chloride-silicate nanowires with diameters of tens of nm up to microns and lengths over 100 μm. As discussed above in Section 3.2 (Cryogenic Measurements), Liang et al.^154^ used these individual nanowires to demonstrate thermometry with a large operating temperature range based on several different NIR ratiometric signals. Beyond the spatial resolution advantage gained from single-particle measurements, other performance improvements and measurements capabilities have also been demonstrated at the single-particle level. Plakhotnik et al.^84^ showed that all-optical ratiometric thermometry based on a single nanodiamond containing approximately 100 NV centers resulted in a noise floor an order of magnitude lower than prior all-optical measurements. Bommidi and Pickel^85^ investigated the temperature-dependent excited state lifetimes of NV centers in individual nanodiamonds and demonstrated single-particle temperature measurements that combine ∼100 nm spatial resolution with ∼100 ns temporal resolution.

### 3.4 Time-Resolved Measurements

There is growing interest in developing thermometry techniques that demonstrate both good spatial and temporal resolution since smaller length scales naturally lead to faster thermal time constants. As a result, studies pertaining to heat dissipation in microelectronics and other nanoscale heating applications can greatly benefit from sensitive thermometry techniques with high spatial and temporal resolution. Luminescence lifetime thermometry measurements are one of the more common time-resolved techniques, given the signal’s robustness to variations in parameters such as particle size, morphology, and emitter concentration.^163^ The lifetimes of most emitters decrease with increasing temperature due to the higher probability of phonon-assisted, nonradiative decay processes at elevated temperatures. Lifetime measurements typically involve a pulsed excitation laser and a time-gated photon monitor such as a camera or a photodetector.

Lifetime thermometry has been demonstrated using a number of UCNP compositions, including NaYF_4_:Yb^3+^,Er^3+^,^53,164−166^ other Yb^3+^ and Er^3+^ codoped hosts,^165,167,168^ and compositions involving other dopants such as Tm^3+^ and Ho^3+^.^163,169^ Because the transitions that give rise to UCNP luminescence are parity forbidden, UCNPs have long lifetimes on the order of hundreds of μs to ms, limiting the temporal resolution of UCNP-based lifetime thermometry. Luminescence lifetime thermometry using Mn^3+^ and Mn^4+^ codoped^170^ and Cr^3+^-doped^163^ nanoparticles with lifetimes on the order of ms and tens of ms, respectively, has also been reported. Other emitters intrinsically have much faster lifetimes, enabling higher temporal resolution measurements. For example, some QDs have temperature-sensitive lifetimes on the order of tens of ns.^171^ As noted previously, Bommidi and Pickel measured lifetimes on the order of tens of ns for NV centers in single nanodiamonds,^85^ complementing prior lifetime measurements of nanodiamond ensembles.^86^ While these techniques are suitable for single-point analysis, measuring lifetimes by scanning across a sample takes considerably longer.^20^ To circumvent this challenge, several groups have developed wide-field imaging techniques to reduce the measurement time.^20,172^ Liu et al.^20^ explored the lifetimes of core/shell NaGdF_4_:Yb^3+^,Er^3+^/NaGdF_4_ UCNPs using single-shot photoluminescence lifetime imaging thermometry (SPLIT), enabling temperature mapping at a video rate of 20 Hz with 20 μm spatial resolution for a 1.5 × 1.5 mm^2^ field of view. Yakunin et al.^172^ combined the highly temperature-sensitive, ns-level lifetimes of low-dimensional tin-halide perovskites with time-of-flight sensors to demonstrate video-rate thermography. Very recently, Li et al.^173^ also reported highly sensitive lifetime-based thermometry using zero-dimensional Te^4+^-doped scandium-halide perovskites with μs-level lifetimes.

Another category of time-resolved techniques relies on the “real-time” monitoring (often referring to integration times on the order of hundreds of ms) of other common thermometry signals, including ratiometric^174−178^ and ODMR signals.^179,180^ Piñol et al.^176^ developed composite nanoparticles containing iron oxide cores for magnetic induction heating functionalized with Eu^3+^ and Tb^3+^ complexes for ratiometric thermometry. The temperature of the nanoparticles was monitored with an integration time of 250 ms as AC magnetic field heating was switched on and off. Caixeta et al.^174^ embedded GeO_2_-Ta_2_O_5_:Yb^3+^,Er^3+^ UCNPs in poly(methyl methacrylate) films and performed ratiometric thermometry measurements with a 200 ms integration time that were in good agreement with simultaneous measurements from a thermocouple. Chen et al.^175^ combined a ratiometric signal based on the Stokes and anti-Stokes fluorescence originating from SiV and GeV centers, respectively, in nanodiamonds with a parallel detection scheme relying on two photodetectors to demonstrate thermometry with a 200 ms integration time. Notably, Tzeng et al.^179^ achieved ODMR thermometry using NV centers in nanodiamonds with temporal resolution better than 10 μs by monitoring the fluorescence intensity at only three frequencies rather than acquiring the entire ODMR spectrum. Yun et al.^180^ later used a related six-frequency measurement approach to demonstrate thermometry with approximately 50 ns temporal resolution. Other work has taken advantage of time-resolved thermometry signals for purposes beyond increased temporal resolution. For example, Qiu et al.^113^ constructed a ratiometric signal based on PbS QDs with ns lifetimes and NaYbF_4_:Tm^3+^ UCNPs with μs lifetimes by separating their spectrally overlapping emission in the time domain.

**Figure 3:**
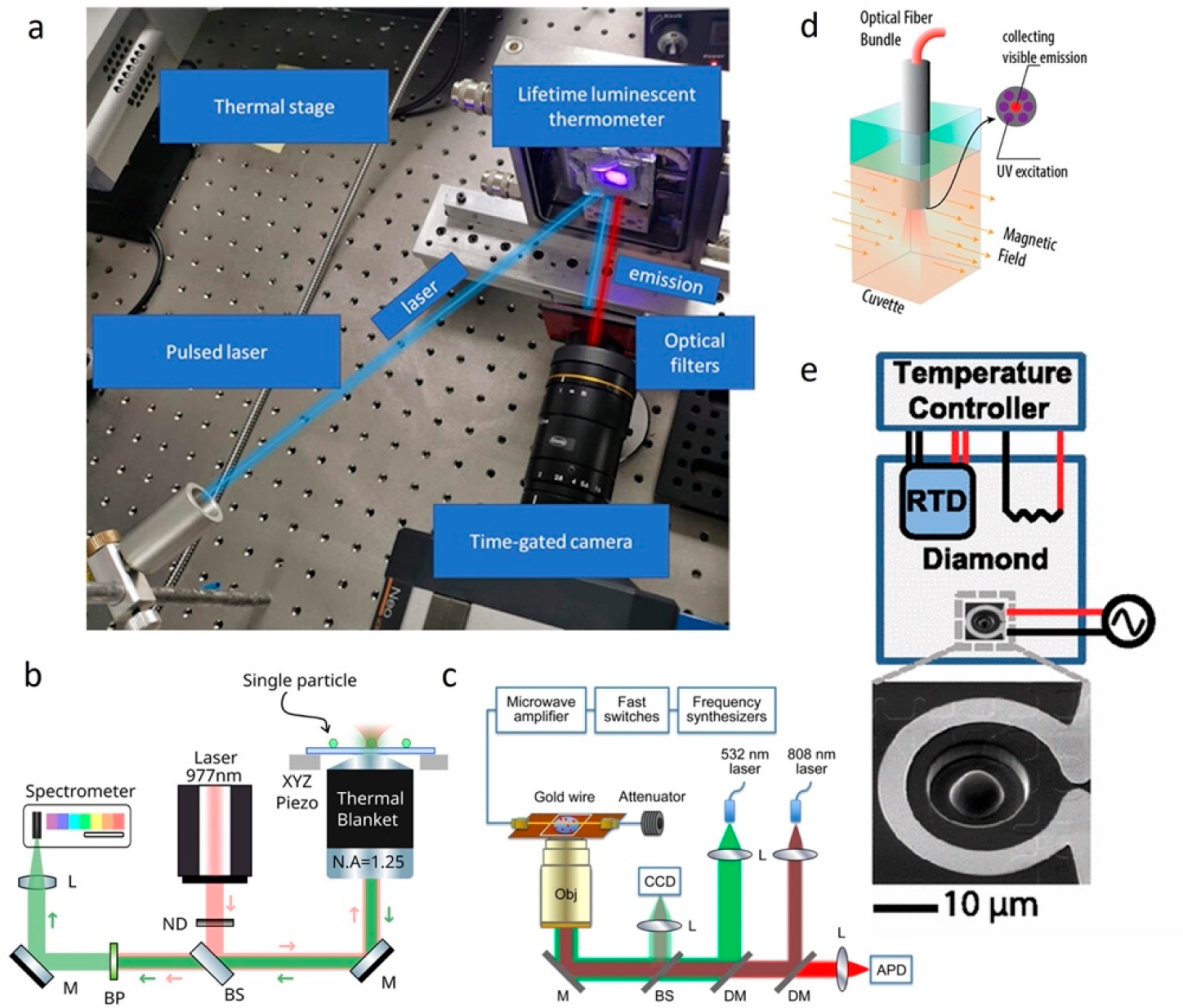
(a) Major components for a time-resolved luminescence thermometry measurement. Reprinted with permission under a Creative Commons CC BY 4.0 license from ref (163) Copyright 2021 John Wiley and Sons. (b) Schematic of a single-particle luminescence thermometry measurement utilizing a spectrometer to record luminescence spectra. Reprinted with permission from ref (66) Copyright 2022 American Chemical Society. (c) Experimental setup including microwave sources for ODMR thermometry measurements. Reprinted with permission from ref (179) Copyright 2015 American Chemical Society. (d) Optical fiber technique used to perform luminescence thermometry measurements for particles dispersed in water. Reprinted with permission from ref (176) Copyright 2015 American Chemical Society. (e) Example experimental setup for high-temperature luminescence thermometry measurements using a solid immersion lens, integrated resistive heater, resistive temperature detector (RTD), and temperature controller. Reprinted with permission under a Creative Commons CC BY 3.0 license from ref (79) Copyright 2012 American Physical Society.

Many of the measurement capabilities described within this section and elsewhere in this review have specific instrumentation requirements. Figure 3 provides a graphical overview of custom imaging and spectroscopy systems that different researchers have constructed to perform luminescence thermometry measurements. As noted above, time-resolved luminescence thermometry measurements often involve pulsed lasers and required photodetectors or cameras with time gating capabilities (Figure 3a). Single-particle temperature measurements frequently require spectrometers coupled with highly sensitive charged-coupled device (CCD) sensors to detect weak emission signals (Figure 3b). ODMR thermometry requires a microwave source in addition to standard luminescence measurement components (Figure 3c). Optical fiber-based measurements can provide a convenient alternative to free-space excitation and collection of luminescence signals (Figure 3d). Measurements performed far from room temperature often require special heating or cooling stages and temperature control systems, with one example system for high-temperature measurements shown in Figure 3e.

## 4 Applications

### 4.1 Electronics and Other Devices

The continued miniaturization of electronics and other devices has exhausted the spatial resolution of many existing thermometry techniques.^101^ Traditional probes with limited spatial resolution, such as thermocouples (∼100 μm)^101^ and IR microscopy (∼5 μm),^181^ cannot resolve temperature gradients or hotspots with smaller characteristic length scales. Many of the luminescence thermometry techniques discussed thus far have been applied to elucidate local temperature distributions in microelectronics and related devices.

**Figure 4:**
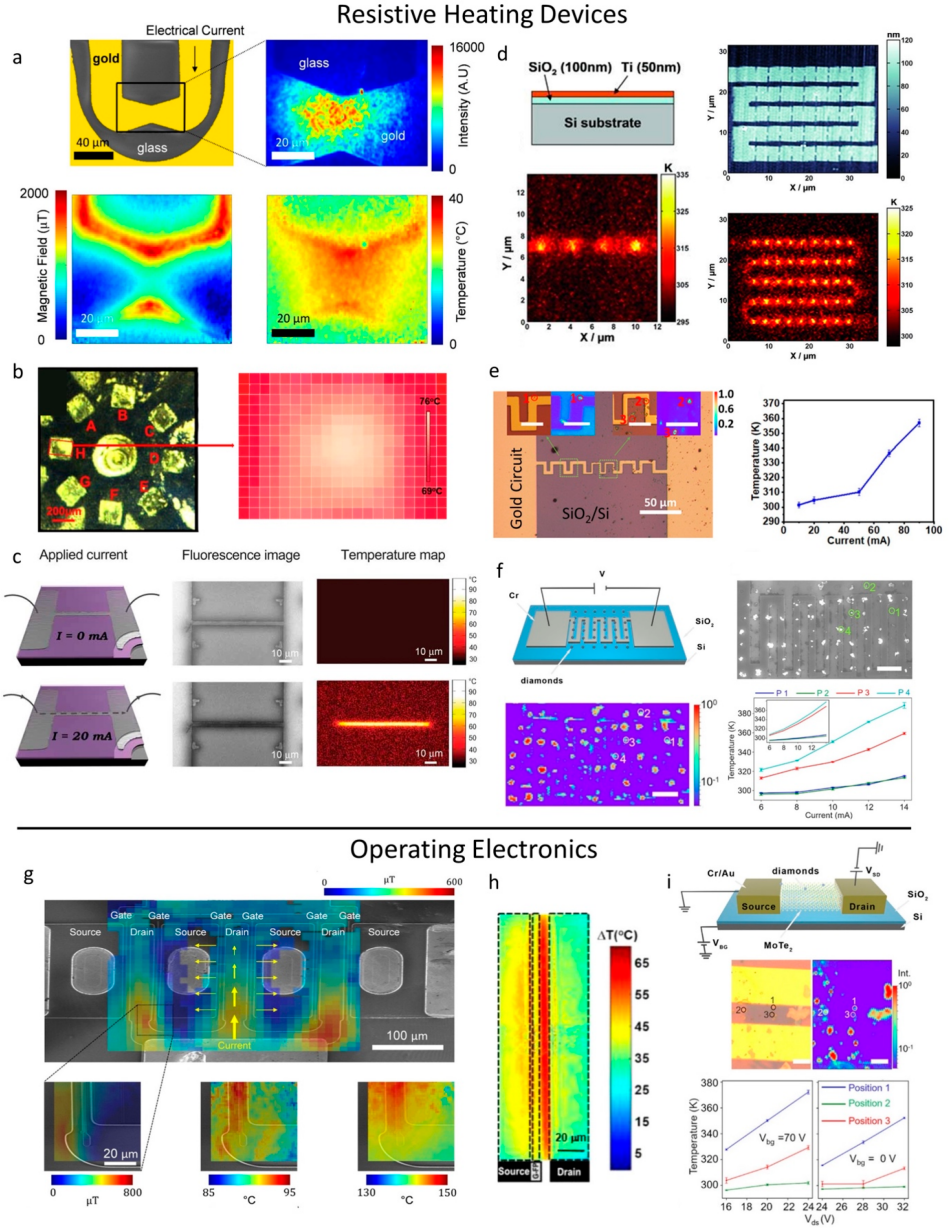
(a) Microfabricated structure imaged via false-color SEM and fluorescence microscopy. NV center-containing nanodiamonds with both temperature- and magnetic field-dependent responses were imaged across the surface. Reprinted with permission from ref.^80^ Copyright 2020 American Chemical Society. (b) A film of ZnCuInS/ZnSe/ZnS QDs was deposited over resistors attached to a printed circuit broad and the QD temperature response was mapped. Copyright IOP Publishing. Reproduced with permission from ref (111), all rights reserved. (c) The temperature rise due to an applied current was measured along a Ni line using the temperature-dependent fluorescence intensity of Rhodamine B. Reprinted with permission from ref (181) Copyright 2008 Wiley-VCH Verlag GmbH & Co. KGaA, Weinheim. (d) An AFM tip with an attached luminescent nanocrystal was scanned across the surface of a Joule-heated structure with repeated constrictions to produce a temperature map. Reprinted with permission from ref (16) Copyright 2011 WILEY-VCH Verlag GmbH & Co. KGaA, Weinheim. (e) The temperature rise of a microcircuit was monitored via the temperature-dependent ZPL shift of color centers in hexagonal boron nitride flakes. Reprinted with permission from ref (183) Copyright 2020 American Chemical Society. (f) Nanodiamonds codoped with SiV and GeV centers were deposited onto a microfabricated structure. The temperature rise of the sample at four different points was measured based on the temperature response of the nanodiamonds and compared to a simulation. Reprinted with permission from ref (175) Copyright 2023 American Chemical Society. (g) The same NV center-containing nanodiamonds from (a) were used to produce temperature and magnetic field maps of the source-gate-drain region of a GaN HEMT. Reprinted with permission from ref (80) Copyright 2020 American Chemical Society. (h) The surface temperature profile of a GaN HEMT was measured using the temperature response of CdSe/CdS quantum rods. Reprinted with permission from ref (105) Copyright 2020 American Chemical Society. (i) The same nanodiamonds from (f) were used to measure the temperature rise at three different positions along an operating 2D MoTe_2_ flake-based FET under various drain-source voltages. Reprinted with permission from ref (175) Copyright 2023 American Chemical Society.

One common strategy for demonstrating the ability to measure local temperature rises resulting from electrical heating is to Joule heat microfabricated metal structures or metallic wires (Figure 4a–f). Löw et al.^181^ used the temperature-dependent fluorescence intensity of Rhodamine B to produce surface temperature maps of a 2 μm wide, 80 μm long Ni line for different input current values. The Rhodamine B fluorescence intensity was quenched on and near the Ni line due to the local temperature increase, and stronger fluorescence quenching was observed for higher currents that produced larger temperature changes. In another example, two Joule-heated Au microwires 25 μm in diameter buried within a polymer film were studied.^182^ Both the steady-state temperature rise due to DC electrical heating and the time-dependent temperature response following pulsed heating were measured at different locations near the microwires using the ZPL shift of NV center-containing nanodiamonds embedded in the film.

In addition to Joule-heated metal lines and wires, more complicated geometries and materials have also been studied.^16,54,80,183−185^ Van Swieten et al.^54^ used a Mo spiral microheater embedded in a Si_3_N_4_ membrane. By drop casting a layer of NaYF_4_:Yb^3+^,Er^3+^ UCNPs approximately several μm in thickness onto the surface, the surface temperature was found to be homogeneous up to the edge of the microheater. An important finding on luminescence thermometry measurement artifacts from this work will be discussed later in Section 5.1 (Measurement Artifacts). Mi et al.^184^ also used sandwich-structured NaYF_4_:Yb^3+^,Nd^3+^/NaYF_4_/NaYF_4_:Yb^3+^,Er^3+^ upconverting nanorods to study the temperature rise at different positions along a Joule-heated magnetoresistive device. Another study mapped the temperature of several resistors in series on a printed circuit board surface using the temperature-dependent PL intensity of core/shell/shell ZnCuInS/ZnSe/ZnS QDs and compared the results to readings from an infrared (IR) thermal camera.^111^ In many cases, the temperature response of the structure under consideration is also calculated analytically or numerically, allowing discrepancies between the measured and modeled temperature profiles to be investigated.^16,80,101,105,181^ In one study, the temperature profile of an Al microelectromechanical system (MEMS) heater was measured using CdSe QDs and calculated using a 1D electrothermal model.^101^ The experimental and modeled results were found to be in good agreement other than at the ends of the heater lines, where the measured temperature profiles indicated that the contact pads could heat above the ambient temperature, whereas the model assumed the pads remained at the ambient value.

In contrast with structures engineered to produce localized Joule heating, other work has studied devices where localized heating is a byproduct of device operation (Figure 4g–i). Such studies are motivated by technologies including microelectronics and optoelectronics where thermal management is required to mitigate local hotspots that can induce performance degradation and failure in operating devices. Chen et al.^175^ used SiV and GeV centers in nanodiamonds to measure the temperature rise at three surface positions of an operating 2D MoTe_2_ flake-based field-effect transistor (FET). The region near the metal-semiconductor junction experienced a large temperature rise of 44 K when the drain-source voltage was increased from 16 to 24 V, which was attributed to the Schottky barrier at the junction. Öner et al.^105^ used the temperature-dependent spectral shift of emission from CdSe/CdS quantum rods to develop a temperature mapping technique called hyperspectral quantum rod thermal imaging (HQTI). Using HQTI, Öner et al. mapped the temperature rise of the passivation layer and source-drain metallization on the surface of a GaN high-electron mobility transistor (HEMT). The HQTI method revealed a temperature rise along the channel that was greater than at the surrounding metal contacts as expected, but no local hotspots or large thermal gradients within the channel were observed. Foy et al.^80^ used ODMR measurements of ∼100 nm thick films of NV center-containing nanodiamonds to demonstrate simultaneous magnetic field and temperature mapping in a wide-field modality at high frame rates of 100 to 1000 Hz. Applying this technique to a GaN HEMT, Foy et al. measured a localized temperature rise near the gate and were also able to resolve a steep temperature drop at the end of the gate. Andrich et al.^186^ developed a fabrication process to create ordered arrays of nanodiamonds containing NV centers in a polymer matrix and used ODMR thermometry to demonstrate temperature mapping of an operating Au coplanar waveguide. Finite element simulations with and without the polymer layer confirmed that the presence of the low thermal conductivity polymer layer did not alter the waveguide temperature profile. Given the numerous applications particularly for Si-based devices, Rodrigues et al.^187^ developed self-assembled monolayers (SAMs) of temperature-sensitive Eu^3+^ and Tb^3+^ complexes that were formed on Si wafers with three different surface coatings, namely, monocrystalline Si, SiO_2_, and polycrystalline Si. Rodrigues et al. also demonstrated that these SAMs could be used to create optical logic gates by taking advantage of bistability in their temperature-dependent ratiometric response.

While temperature mapping can be performed by monitoring many different particles spread over the surface of a device, other methods instead involve scanning a probe with attached emitters across a device. In a foundational demonstration of this concept, a Yb^3+^ and Er^3+^ codoped fluoride glass particle was glued to the end of an AFM tip.^12^ A temperature map was recorded by scanning the particle across a 20 μm wide Joule-heated polysilicon resistor and measuring the ratiometric signal from the particle. Another study measured the temperature of two microheaters using a PbF_2_:Yb^3+^,Er^3+^ nanocrystal on an AFM tip.^16^ Two Ti microheaters with repeated constrictions that increased the local current density were patterned on top of Si/SiO_2_ substrates. One microheater had a 100 nm SiO_2_ layer between the Si and Ti layers, while the other sample had a 1 μm thick layer. Temperature maps of the two devices revealed that the thicker SiO_2_ layer increased the lateral heat spreading and reduced the temperature difference between the local hotspots and the surroundings when compared to the thinner SiO_2_ layer. Laraoui et al.^160^ developed a related approach to map thermal conductivity by mounting a nanodiamond containing an NV center on the end of an AFM tip. An electrical current was used to heat the tip and the ODMR signal from the NV center was used to measure the tip temperature, which changed in response to being brought in contact with surfaces of different thermal conductivities. Thermal conductivity mapping was demonstrated for an 18 nm thick Au structure patterned on a sapphire substrate. The thin-film Au thermal conductivity was measured to be below the bulk value, in accordance with prior measurements using other techniques.

Recognizing the pervasiveness of quick response (QR) codes and their ability to be read using ubiquitous smartphone hardware, Ramalho et al.^188^ developed a luminescent QR code containing Eu^3+^ and Tb^3+^ codoped organic–inorganic hybrid materials with temperature-dependent emission. The green to red intensity ratio served as the temperature-dependent signal and could be detected using a standard smartphone camera. The results agreed well with emission spectra obtained by a more sophisticated spectrometer, important for advancements in the Internet of Things and on-demand thermal probing. In contrast to what has been discussed so far, where luminescent emitters are used to measure the temperature of a heater or device, Martínez et al.^189^ demonstrated the opposite effect, where a device was heated specifically to modulate the emission from two different types of UCNPs varying in both size and shape. Silver nanowires in a poly(methyl methacrylate) layer were Joule heated using Au contacts. NaYF_4_:Yb^3+^,Ln^3+^ and core/shell NaGdF_4_:Yb^3+^,Ln^3+^/NaYF_4_ (Ln = Tm, Er, Ho) UCNPs were deposited on the surface and their emission was selectively quenched or enhanced based on the surrounding temperature. The end result was an electrochromic device where the color output was determined by the local heating of the emitters.

### 4.2 Plasmonics

Plasmonic nanostructures, which are often composed of noble metals, interact resonantly with specific frequencies of light. These interactions lead to strong absorption, scattering, and localization of the electric field, which are known as plasmonic effects. Plasmonic nanostructures have wide-ranging applications in areas including photothermal therapy, solar energy conversion, and surface-enhanced Raman spectroscopy. Plasmonic nanostructures are also often used to enhance luminescence signals. Luminescence thermometry and plasmonic nanostructures can be combined for different purposes, such as thermometry and optical absorption measurements of the plasmonic nanostructures or thermal property measurements of other materials. While applying luminescence thermometry to plasmonic nanostructures requires careful validation, studies of some luminescent thermometers such as NaYF_4_:Yb^3+^,Er^3+^ UCNPs have shown that common thermometry signals remain valid in a plasmonic environment.^164^ Glais et al.^190^ also showed that the temperature-dependent lifetime of Cr^3+^ measured from ZnGa_2_O_4_:Cr^3+^,Bi^3+^ nanoparticles was unaffected by a plasmonic environment.

**Figure 5:**
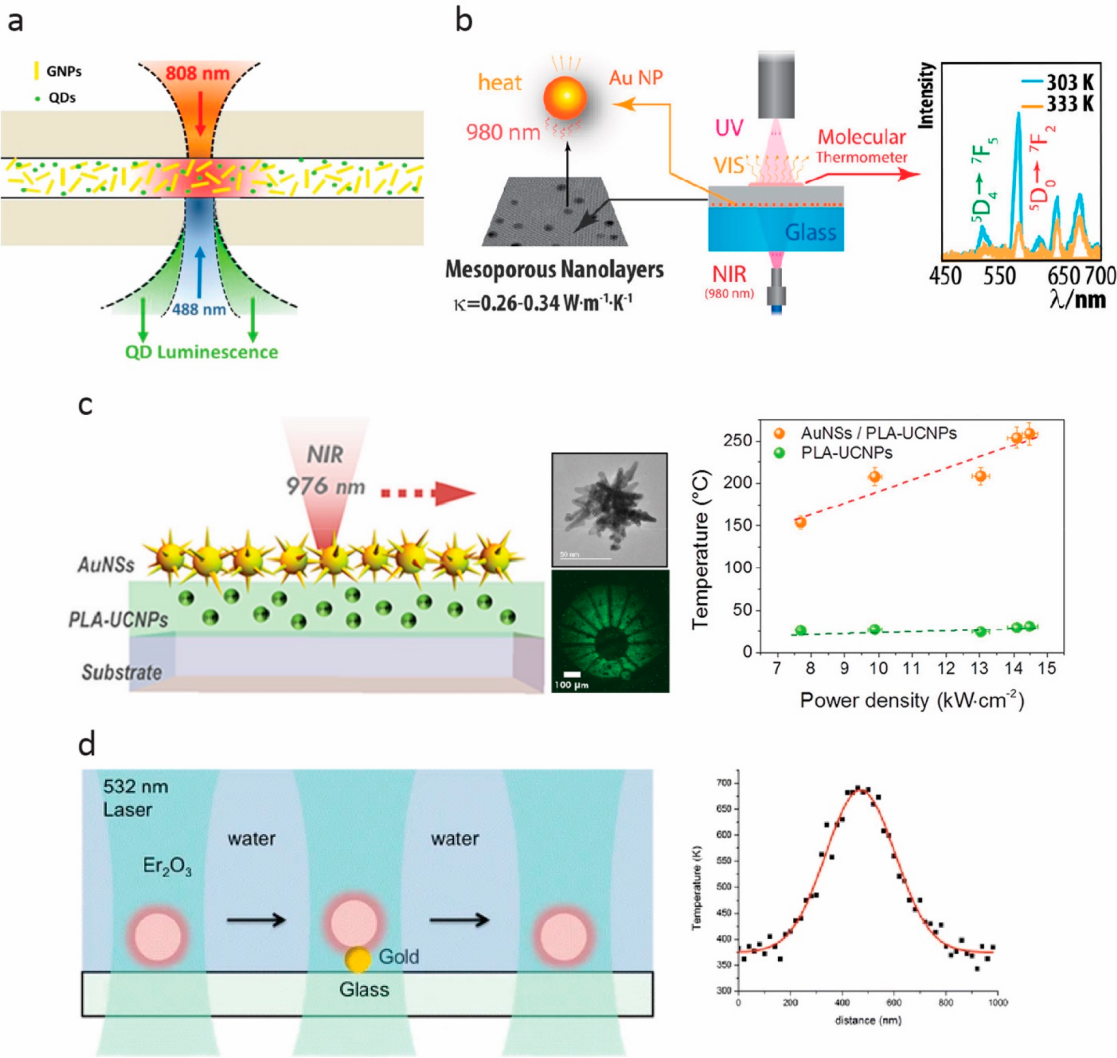
(a) The temperature-dependent luminescence of CdSe QDs excited by a 488 nm laser was used to assess the absorption and heating efficiency of gold nanoparticles (GNPs) heated by an 808 nm laser. Reprinted with permission from ref (102) Copyright 2014 American Chemical Society. (b) Gold plasmonic nanoparticle heaters and Tb^3+^/Eu^3+^ codoped organic–inorganic hybrid thermometers were combined with a thermal model to determine the thermal conductivity of mesoporous nanolayers. Reprinted with permission from ref (191) Copyright 2017 American Chemical Society. (c) A maskless lithography process was developed based on plasmonic gold nanostars (AuNSs) tuned to absorb at the same wavelength as UCNPs used for temperature monitoring. Reprinted with permission from ref (194) Copyright 2019 American Chemical Society. (d) The temperature profile of a gold nanodot and its surroundings was measured by scanning optically trapped Er_2_O_3_ nanoparticle thermometers. Reprinted with permission from ref (196) Copyright 2016, Springer-Verlag Berlin Heidelberg.

Maestro et al.^102^ used QD thermometry to investigate the absorption and heating efficiency of different morphologies of gold nanoparticles (GNPs), namely, nanorods, nanocages, nanoshells, and nanostars (Figure 5a). The GNPs and CdSe QDs were both dispersed in purified water and excited by an 808 nm laser and a 488 nm laser, respectively. The GNP concentration was high enough to introduce a detectable temperature rise, while the QD concentration was low enough to avoid additional, non-negligible optical absorption. After measuring the extinction cross-sections via optical transmission experiments and the temperature rise at different excitation powers via QD thermometry, the heating efficiency and the absorption and scattering cross-sections of the GNPs were obtained by combining the experimental results with a thermal model. In addition to measuring the optical and thermal properties of plasmonic nanostructures, the combination of a plasmonic heat source and luminescence thermometry can be applied to measure the thermal properties of other materials, as demonstrated by Brites et al.^191^ Tb^3+^ and Eu^3+^ codoped organic–inorganic hybrid luminescent thermometers were deposited on top of silica and titania mesoporous nanolayers, while gold nanoparticles for plasmonic heating were deposited at the bottom of the silica mesoporous nanolayers or in the pores of the titania mesoporous nanolayers (Figure 5b). By combining the experimental temperature measurements with a thermal model, the thermal conductivities of the silica and titania mesoporous nanolayers were obtained. The calculated values were similar to previously reported literature values. This method also avoids the need to deposit a metal heater line on the sample surface, which is required by approaches such as the 3ω method.

Many reports of luminescence thermometry applied to plasmonic nanostructures use a combination of two lasers operating at different wavelengths. Experiments that use only one laser to excite both the plasmonic nanostructures and the luminescent thermometers are possible but require careful selection or design of the plasmonic nanostructures and luminescent thermometers. A one-beam system can overcome two potential disadvantages of a two-beam system: additional plasmonic heating due to the laser used to excite the luminescent thermometers and artifacts in the temperature measurements introduced by the laser used to excite the plasmonic nanostructures. However, the plasmonic absorption and luminescent thermometer excitation wavelengths must overlap to obtain the temperature readout and plasmonic heating simultaneously. With only one beam, Maestro et al.^13^ measured the temperature rise of an optical plasmonic recording medium, which contained CdSe QDs serving as luminescent thermometers and gold nanorods acting as plasmonic heat sources. Using two-photon excitation of the CdSe QDs, an 800 nm laser was able to excite both the gold nanorods and QDs. Taking a different approach, Rohani et al.^192^ tuned the plasmonic resonance of gold nanorods to match the 980 nm absorption wavelength of NaGdF_4_:Yb^3+^,Er^3+^ UCNPs and demonstrated both plasmon-enhanced UCNP luminescence and a measured temperature rise of approximately 160 °C. Debasu et al.^193^ combined (Gd,Yb,Er)_2_O_3_ nanorods with GNPs and showed that although the GNP absorbance was stronger in other spectral regions, it was sufficiently large at the 980 nm nanorod excitation wavelength to generate temperature rises of hundreds of degrees. Martínez et al.^194^ deposited gold nanostars (AuNSs) on top of a polylactic acid (PLA) film, which has a glass transition temperature near 60 °C, embedded with NaGdYbErF_4_/NaGdF_4_ core/shell UCNPs (Figure 5c). The AuNS synthesis parameters were adjusted to match their plasmonic resonance to the 976 nm UCNP laser excitation wavelength. When locally heated above the PLA glass transition temperature, the AuNSs attach to the PLA surface and the unexposed PLA can subsequently be dissolved using acetone, behavior that was harnessed to develop a maskless lithography process. By sharing the same excitation laser, the UCNPs were able to probe the temperature rise resulting from plasmonic heating of the AuNSs and provide real-time monitoring of the lithography process. Huang et al.^195^ instead tuned the plasmonic resonance of gold nanorod cores surrounded by upconverting NaYF_4_:Yb^3+^,Er^3+^ shells to ∼ 650 nm to match the red Er^3+^ emission from the ^4^F_9/2_ excited state. The gold nanorod cores generated heat upon absorbing this red upconverted emission, while the green upconverted Er^3+^ emission was used for thermometry.

Although most measurements characterize the temperature rise resulting from the collective contributions of many individual plasmonic nanoparticles, Baral et al.^196^ reported an approach to measure the temperature profile near a single isolated gold nanostructure using the temperature-dependent emission from a small cluster of optically trapped Er_2_O_3_ nanoparticles (Figure 5d). Er_2_O_3_ nanoparticles ∼45 nm in size were suspended in water, optically trapped, and scanned to map the temperature rise of a gold nanodot 100 nm in diameter and its surroundings. From the measured temperature profile, the point spread function of the thermometer was estimated to be approximately 165 nm, suggesting that a small cluster of Er_2_O_3_ nanoparticles was trapped. While trapping and stabilizing a single Er_2_O_3_ nanoparticle of this size is challenging due to the weak optical forces in the case of laser trapping of small nanoparticles, trapping a cluster nonetheless allowed for temperature measurements with subdiffraction limited spatial resolution.

### 4.3 Catalysis

Catalysis improves chemical reaction rates by providing reaction pathways with lower activation energy barriers and is essential to critical industrial processes such as ammonia production, emerging green technologies like hydrogen production and carbon dioxide reduction, and myriad other areas. Temperature plays a fundamental role in catalysis, motivating the need for in situ temperature measurements during catalytic reactions. Reaction rates are generally improved by increasing the temperature, which can be understood from the Arrhenius equation. Common thermometry techniques such as thermocouples, nuclear magnetic resonance methods, and IR thermography have several drawbacks that include spatial resolution, complicated data analysis procedures, and requiring previous knowledge of the emissivity of the target surface. Meanwhile, luminescence thermometry has numerous advantages, such as enabling remote detection, requiring simpler calibration, and having high spatial resolution.

**Figure 6:**
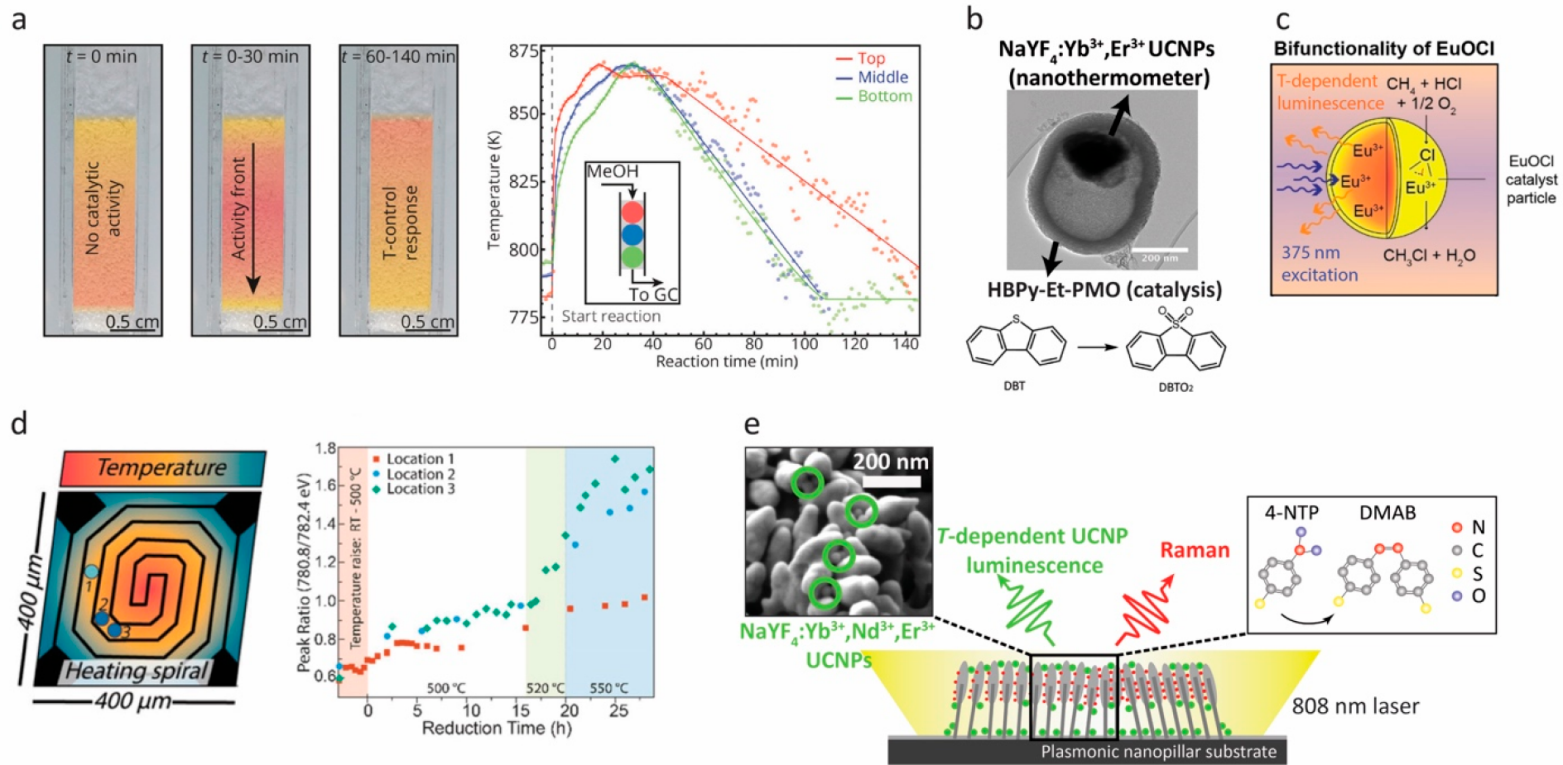
(a) The temperature at the top, middle, and bottom of a fixed-bed reactor was monitored using NaYF_4_:Yb^3+^,Er^3+^ microcrystals mixed with a solid catalyst during the methanol-to-hydrocarbons reaction, revealing a moving temperature front. Reprinted with permission from ref (15) Copyright 2018 American Chemical Society. (b) NaYF_4_:Yb^3+^,Er^3+^ nanothermometers were grown inside hollow bipyridine-ethane periodic mesoporous organosilica hosts to avoid catalytic activity loss that can result from blocking catalyst surface area with nanothermometer materials. Reprinted with permission from ref (200) Copyright 2022 American Chemical Society. (c) EuOCl was applied as both a catalyst and thermometer for the exothermic methane oxychlorination reaction. Reprinted with permission under a Creative Commons CC BY 4.0 license from ref (203) Copyright 2022 John Wiley and Sons. (d) Microcrystalline NaYF_4_:Yb^3+^,Er^3+^ thermometers revealed temperature gradients and deviations from the set temperature on MEMS reactors. Reprinted with permission from ref (204) Copyright 2019 The Authors. Published by Wiley-VCH Verlag GmbH & Co. KGaA. (e) A single 808 nm laser was used to both photocatalyze a chemical reaction and excite NaYF_4_:Nd^3+^,Yb^3+^,Er^3+^ UCNP thermometers, enabling simultaneous temperature and reaction monitoring during plasmonic photocatalysis. Reprinted with permission from ref (206) Copyright 2023 Wiley-VCH GmbH.

Different methods have been used to combine luminescent thermometers and catalytic materials. A convenient method is to physically mix the luminescent thermometers with the catalyst.^15,197^ Geitenbeek et al.^15^ mixed NaYF_4_:Yb^3+^,Er^3+^ microcrystals with a commercial solid catalyst, zeolite H-ZSM-5, to measure temperature in a fixed-bed reactor (Figure 6a). The temperature as a function of time at the top, middle, and bottom of the reactor bed was monitored during the methanol-to-hydrocarbons (MTH) reaction by using a fiber probe to collect luminescence spectra from the NaYF_4_:Yb^3+^,Er^3+^ particles at three different heights. Meanwhile, several different types of hydrocarbons that form as reaction products were measured using online gas chromatography. Although the temperature uncertainty increased from approximately 0.3 to 22 K over the course of the reaction due to reduced luminescence intensity, the NaYF_4_:Yb^3+^,Er^3+^ particles measured temperatures up to nearly 875 K, indicating excellent thermal stability. The temperature increases measured in the system matched the exothermic nature of the MTH reaction, and a moving temperature front observed during the reaction was also consistent with prior reports of coke deposition.

Another strategy is to realize the dual functions of thermometer and catalyst in a composite material. Jena et al.^198^ created nanocomposites consisting of Yb^3+^ and Er^3+^ codoped or Yb^3+^ and Tm^3+^ codoped UCNPs combined with UiO-66-NH_2_ MOFs. The presence of the UCNP thermometers did not prevent catalytic activity during the esterification of lauric acid with methanol, although a longer reaction time was required than for pristine MOFs due to the reduction in available surface area. The temperature-dependent UCNP spectral features also remained effective for thermometry. Krishnaraj et al.^199^ developed composites consisting of covalent organic frameworks (COFs) grown around UCNPs and subsequently grafted with Cu ions for catalysis, an architecture that avoided any loss of COF surface area. Similarly, Sun et al.^200^ grew NaYF_4_:Yb^3+^,Er^3+^ nanothermometers inside hollow bipyridine-ethane periodic mesoporous organosilica hosts to avoid catalytic activity loss (Figure 6b).

In some cases, the luminescent thermometer itself can serve as a catalyst. Kaczmarek et al.^201^ grafted lanthanide ions to covalent organic frameworks (COFs), enabling simultaneous temperature measurements and catalysis using a single material. Gomez et al.^202^ demonstrated the synthesis, temperature sensing, and catalytic properties of a series of lanthanide MOFs based on lanthanide ions and 2-phenylsuccinate. The MOFs were able to catalyze a cyanosilylation reaction (CSR), suggesting the possibility of using these materials to investigate the influence of temperature on CSRs. In another example reported by Terlingen et al.,^203^ EuOCl served as both a thermometer and catalyst for the exothermic methane oxychlorination reaction (Figure 6c). A temperature rise of 16 K over the oven temperature was recorded due to the exothermic nature of the reaction, while no measurable temperature gradient was observed between the top and bottom of the reactor.

Beyond measuring the temperature at selected locations along a reactor bed, incorporating microscopy capabilities can enable higher spatial resolution temperature mapping to correlate the temperature and reaction activity more precisely. Van Ravenhorst et al.^204^ mapped the temperature distribution in a 300 μm MEMS reactor with approximately 10 μm resolution (Figure 6d). Rather than monitoring the temperature and reaction simultaneously, microcrystalline NaYF_4_:Yb^3+^,Er^3+^ particles for thermometry and Co/TiO_2_ catalyst particles were deposited on separate MEMS reactors. A 200 °C temperature gradient between the center and edge of the reactor was measured in vacuum, consistent with separate scanning transmission X-ray microscopy measurements that showed variations in catalytic activity for individual catalytic particles at different locations on the reactor surface. Additional luminescence thermometry measurements performed in air and in the presence of flowing gases showed deviations from the set reactor temperature, suggesting that accurate temperature measurements can play a key role in optimizing the catalyst and reactor design.

In addition to online gas chromatography or mass spectrometry, surface-enhanced Raman spectroscopy is a powerful tool for monitoring chemical bond formation and can offer detailed information about the reaction process. Because NIR-excited Raman signals remain within several hundred nm of their excitation wavelength while NIR-excited UCNP luminescence is typically anti-Stokes shifted to the visible wavelength range, UCNP thermometry and Raman-based reaction monitoring can be performed concurrently. Hartman et al.^205^ developed multifunctional sensors consisting of shell-isolated nanoparticles for Raman signal enhancement, core/shell NaYF_4_:Yb^3+^/Er^3+^ UCNPs for luminescence thermometry, and Rh catalysts, allowing for operando thermometry and reaction monitoring at the single catalyst particle level. A 980 nm laser was used to excite the UCNPs and a 785 nm laser was used for Raman spectroscopy. The luminescence thermometry measurements revealed differences between the set and measured temperatures that were attributed to heat dissipation into the flowing gas, with a higher thermal conductivity gas resulting in a larger discrepancy. Ye et al.^206^ combined UCNP thermometry and Raman-based reaction monitoring to investigate thermal contributions to plasmonic photocatalysis (Figure 6e). Here, a single 808 nm laser was used to both photocatalyze the chemical reaction and excite NaYF_4_:Nd^3+^,Yb^3+^,Er^3+^ UCNP thermometers. Local laser-induced temperature rises over 40 K were recorded, yet heating alone could not catalyze the reaction, indicating the potential of luminescence thermometry to further elucidate plasmonic photocatalysis mechanisms.

### 4.4 Optical Trapping and Levitation

**Figure 7:**
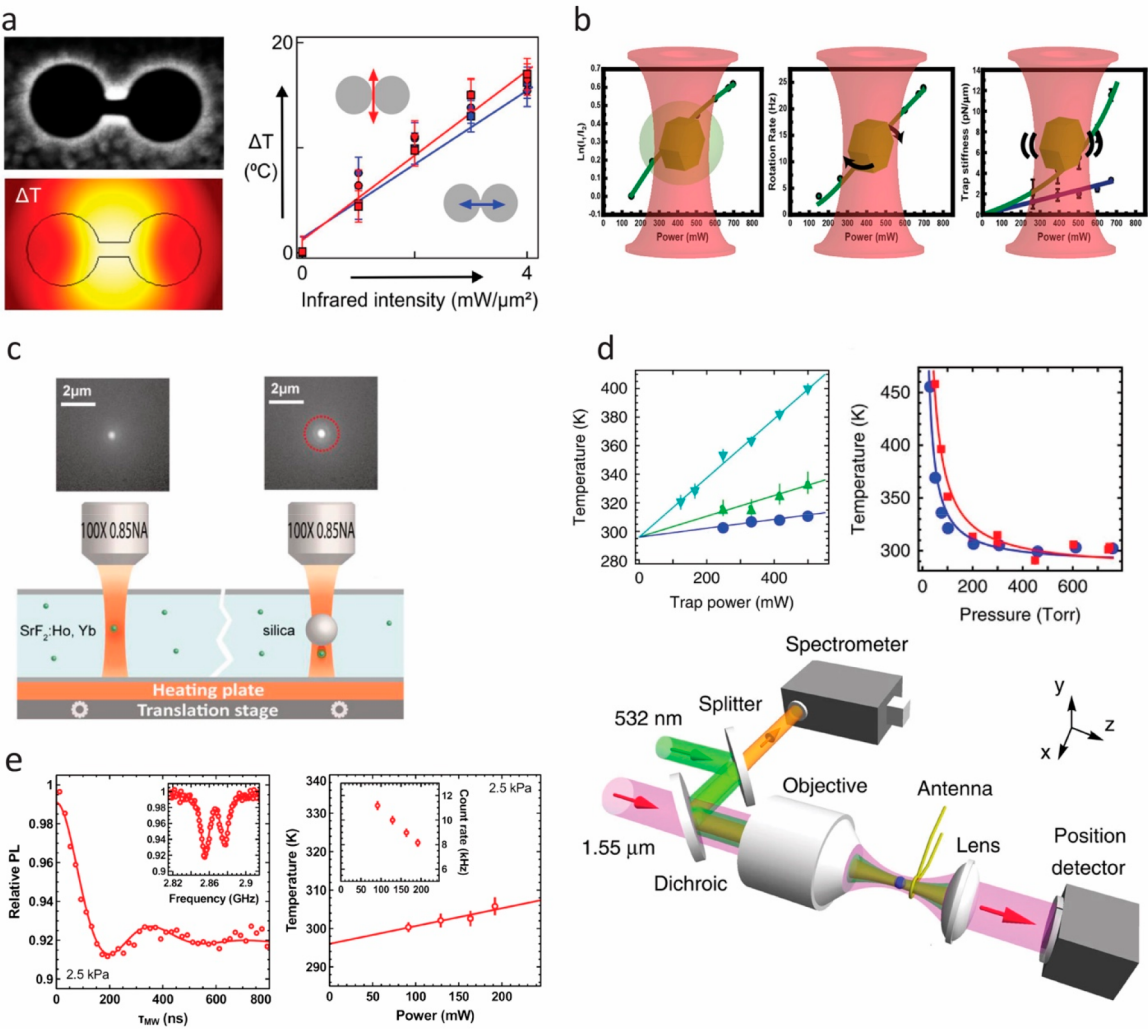
(a) Three different temperature-dependent fluorescence signals from Alexa Fluor 647 were applied to measure the laser-induced temperature rise in plasmonic nanohole structures used for optical trapping. Reprinted with permission from ref (208) Copyright 2019 American Chemical Society. (b) The luminescence intensity ratio, optical rotation rate, and trap stiffness were all used to measure the temperature of an optically trapped microscale upconverting particle, allowing the measured internal and center-of-mass temperatures to be compared. Reprinted with permission from ref (210) Copyright 2018 American Chemical Society. (c) A silica microsphere was used to focus the trapping laser below the diffraction limit and create a “photonic nanojet,” enabling stable trapping of a single sub-10 nm UCNP in water from 20 to 90 °C. Reprinted with permission from ref (212) Copyright 2021 Wiley-VCH GmbH. (d) ODMR thermometry based on NV centers was used to measure the temperature rise of an optically levitated nanodiamond in low vacuum due to laser absorption by impurities, which exceeded 150 K. Reprinted with permission under a Creative Commons CC BY 4.0 license from ref (216) Copyright 2016 Springer Nature. (e) ODMR thermometry was used to demonstrate that optically levitated nanodiamonds containing single NV centers remained close to room temperature even in low vacuum. Reprinted with permission from ref (218) Copyright The Optical Society.

In optical trapping, the power density of the trapping laser must be sufficiently high for stable trapping of micro to nanoscale targets, making it possible to induce an unwanted temperature rise. This undesirable heating can damage optically trapped targets or increase their Brownian motion, resulting in loss of the target. Therefore, quantitative investigation is required to assess the temperature rise of trapped objects. Fluorescence correlation spectroscopy was applied to quantify local heating in different solutions (ethylene glycol, ethanol, and water) under optical trapping conditions over a decade ago.^207^ Jiang et al.^208^ later used three different temperature-dependent fluorescence parameters—the intensity, diffusion time, and lifetime of Alexa Fluor 647— to study the local temperature rise due to a 1064 nm trapping beam focused on single and double nanoholes milled in gold films, which are plasmonic nanostructures commonly used for optical trapping (Figure 7a). The temperatures measured using these three independent parameters were in excellent agreement with one another and with numerical simulations, demonstrating that even at a moderate power density of 2 mW μm^–2^ the temperature rise could reach nearly 10 °C.

While laser heating during optical trapping is often considered a parasitic side effect, in other cases it offers fundamental information about heat transfer, Brownian dynamics, and optical forces at small length scales. Rodríguez-Sevilla et al.^209^ used ratiometric luminescence thermometry based on microscale upconverting NaYF_4_:Yb^3+^,Er^3+^ particles trapped in water to investigate the optical spinning behavior of the particles themselves, a capability enabled by their intrinsic birefringence, as a function of laser power. This study enabled the subsequent development of upconverting particle-based mechanical microthermometers with thermal sensitivity two times that of the luminescence-based thermometry. In another study, Rodríguez-Sevilla et al.^210^ used three different signals to measure the temperature of optically trapped microscale upconverting particles in water, namely, the luminescence intensity ratio, the optical rotation rate, and the trap stiffness, facilitating comparison of the measured internal and center-of-mass temperatures (Figure 7b). Lu et al.^211^ also measured the temperature-dependent diffusive velocity of a single optically trapped NaYF_4_:Yb^3+^,Er^3+^ UCNP and compared their results with the predictions of the Stokes–Einstein relation. Thermometry using optically trapped upconverting particles with nanoscale characteristic dimensions can enable higher spatial resolution measurements, but such measurements are difficult due to the weak optical trapping forces and small luminescent signals. Lu et al.^212^ overcame these challenges by using a silica microsphere to focus the trapping laser below the diffraction limit and create a “photonic nanojet,” which allowed for stable trapping of a single sub-10 nm UCNP in water from 20 to 90 °C (Figure 7c).

Another commonly investigated material for optical trapping and thermal sensing is NV centers in nanodiamonds. Optically levitated nanodiamonds (i.e., nanodiamonds trapped in vacuum or in a gas) are appealing for quantum sensing and metrology and for creating spin-optomechanical hybrid systems.^213^ Heating caused by the trapping laser is often unfavorable since it can lead to quantum decoherence and result in the loss of the nanodiamond from the trap or of the fluorescence signal. Rahman et al.^214^ demonstrated that nanodiamonds levitated in air burn at subatmospheric pressures and graphitize in nitrogen below 10 mbar, which was attributed to absorption of the 1064 nm trapping laser by impurities in the nanodiamonds and amorphous carbon present on their surfaces. Frangeskou et al.^215^ later showed that nanodiamonds created by milling ultrahigh purity diamond displayed no detectable heating below 5 mbar as determined from their center-of-mass motion.

Hoang et al.^216^ demonstrated electronic spin control and ODMR thermometry of NV centers in a nanodiamond that was optically levitated using a 1550 nm laser rather than the common 1064 nm trapping wavelength to increase the percentage of trapped nanodiamonds with a strong fluorescence signal (Figure 7d). In low vacuum, temperature rises over 150 K were measured, which was again attributed to impurities in the nanodiamonds. Delord et al.^217^ recorded ODMR spectra from NV centers in levitated nanodiamonds at pressures down to 0.2 mbar by using a Paul trap rather than an optical dipole trap and showed that the 532 nm laser used for NV center thermometry could induce temperatures rises of tens of degrees for laser powers on the order of tens of μW at these low pressures. Pettit et al.^218^ demonstrated stable optical levitation of nanodiamonds containing single NV centers at both atmospheric pressure and in low vacuum (Figure 7e). ODMR thermometry indicated that the nanodiamond temperature was maintained near room temperature for the range of pressures and trapping powers considered. The transverse spin coherence time was also shown to remain robust. Rivière et al.^219^ used simultaneous ODMR and center-of-mass motion thermometry to study the Brownian motion of NV center-containing nanodiamonds heated by a 1550 nm trapping laser, another example of harnessing laser heating of optically trapped particles for fundamental studies of heat transfer and Brownian dynamics.

### 4.5 Pressure Sensing

Studies of matter at high pressures of tens to hundreds of GPa are relevant across areas including geology, astrophysics, and materials synthesis, motivating the development of methods to access these extreme conditions in a laboratory setting. Tribology studies can likewise involve lubricants that experience pressures in the range of several GPa. These high-pressure environments are often coupled with elevated temperatures. Intuitively, remote detection is advantageous for sensing quantities like pressure and temperature under these conditions. The diamond anvil cell (DAC) is a commonly employed device capable of generating pressures of hundreds of GPa. While luminescence-based pressure sensing is common for DACs, accurate, simultaneous temperature and pressure measurements have remained elusive until recently.^220^ YF_3_:Yb^3+^,Er^3+^ upconverting microparticles were used in a DAC to measure pressures up to ∼8 GPa based on the spectral shift of the red Er^3+^ peak near ∼665 nm due to Stark sublevel position changes caused by increased crystal field splitting at higher pressures; meanwhile, temperature was measured up to ∼473 K via the common ratiometric signal based on the green Er^3+^ emission.^220^ The pressure and temperature responses showed only minor interdependence, and the measured pressure and temperature values were in good agreement with reference measurements from ruby emission and a thermocouple, respectively. Zheng et al.^221^ demonstrated stabilization of the less common divalent oxidation state of Tm (i.e., Tm^2+^) in a SrB_4_O_7_ matrix and used this probe for separate temperature and pressure measurements in a DAC at pressures up to ∼13 GPa. Pressure sensing was performed based on both the spectral shift and broadening of a Tm^2+^ emission peak. Zheng et al. also demonstrated thermometry using four different Tm^2+^ luminescence signals (peak width, spectral peak shift, intensity ratio of two vibronic components in the emission spectra, and lifetime) over a wide temperature range of approximately 10 to 400 K.

Other work on luminescence-based thermometry and pressure sensing conducted within DACs has been motivated by tribology applications involving comparatively modest pressures. Albrahani et al.^222^ calibrated the spectral shift of the emission peak from CdSe/CdS/ZnS core/shell/shell QDs between 295 and 373 K and at pressures up to 1.1 GPa. The peak shifts to shorter wavelengths at higher pressures, in agreement with prior pressure-dependent studies of CdSe. The temperature rise due to shear heating of a liquid film flowing between two parallel plates was also studied. Seoudi et al.^223^ later applied QDs of the same composition to measure the temperature rise and pressure of lubricants in elastohydrodynamic contacts following calibration in a DAC. Recently, Zhou et al.^224^ also used GdVO_4_:Yb^3+^,Er^3+^ UCNPs suspended in an ester-based oil lubricant to demonstrate ratiometric thermometry up to 473 K at pressures up to approximately 1.1 GPa in a DAC, suggesting a path toward tribology applications of UCNP thermometry.

Recent work has also demonstrated the adaptation of proven luminescence thermometry probes for optical pressure sensing in low-pressure and vacuum systems. Runowski et al.^225^ showed that ratiometric thermometry could be used to measure increased laser-induced heating of YVO_4_:Yb^3+^,Er^3+^ UCNPs at vacuum pressures that results from decreased heat dissipation into the surrounding air. The temperature rise can be calibrated as a function of pressure and subsequently used for pressure sensing. A similar strategy was demonstrated using glass microspheres containing Nd^3+^-doped nanoperovskites that act as whispering gallery mode resonators.^226^ In addition to the luminescence intensity ratio and the temperature-dependent spectral shifts and broadening of the Nd^3+^ emission peaks, the temperature dependence of the whispering gallery mode spectral shift was evaluated and found to result in a pressure sensitivity almost twice as high. The techniques discussed thus far have focused on optimizing a pressure-dependent signal for either low- or high-pressure environments. Runowski et al.^227^ demonstrated an upconverting YPO_4_:Yb^3+^,Er^3+^ probe that combines low- and high-pressure sensing capabilities, using laser heating measured by Er^3+^-based ratiometric thermometry to extract low pressures and the pressure-dependent spectral shift of a NIR Er^3+^ peak in the high-pressure range. With this approach, pressures spanning 9 orders of magnitude, from 10^–8^ GPa to 10 GPa, could be determined.

### 4.6 Microfluidics and Nanofluids

**Figure 8:**
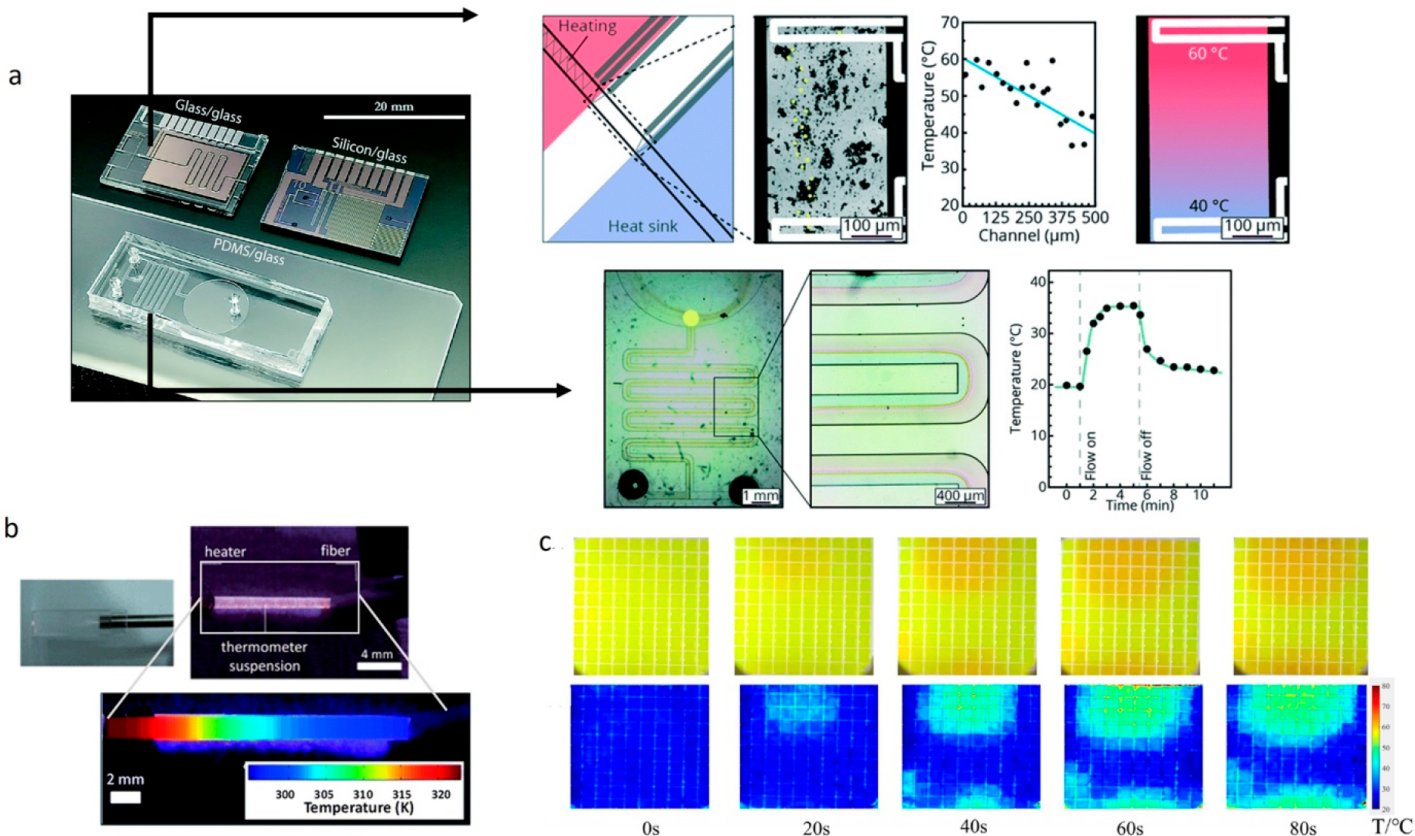
(a) Several examples of microfluidic devices studied under various conditions using the temperature-dependent luminescence from NaYF_4_:Yb^3+^,Er^3+^ UCNPs. A glass/glass microfluidic device was used to study the temperature profile that developed across a fluid channel with one heated side, and a PDMS/glass device was used to observe temperature changes due to the mixing of reactants as part of an exothermic reaction. Reproduced from ref (229) with permission from the Royal Society of Chemistry. (b) Temperature mapping of a nanofluid containing Eu^3+^/Tb^3+^ codoped particles. Reproduced from ref (230) with permission from the Royal Society of Chemistry. (c) Color images of an ethanol solution containing microwave-heated thermochromic zinc-based MOFs, which were used to generate temperature maps of the fluid across different heating times. Reprinted with permission from ref (234) Copyright 2022 Elsevier, Ltd. All rights reserved.

While many thermal studies of microfluidic devices are biologically motivated, other motivating applications include evaluating the performance of microchannel heat sinks, optimizing nanomaterial synthesis procedures, and monitoring chemical reactions. Chamarthy et al.^228^ used a ratiometric technique based on Rhodamine B and Rhodamine 110 fluorescence to map temperature nonuniformities in a silicon microchannel heat sink resulting from flow maldistribution. Geitenbeek et al.^229^ fabricated microfluidic devices with different material compositions and performed temperature measurements using the ratiometric signal from NaYF_4_:Yb^3+^,Er^3+^ UCNPs dispersed in fluids that were in good agreement with readings from platinum resistance temperature detectors (RTDs) (Figure 8a). Unlike RTDs, which can only provide temperature readings at fixed locations, UCNPs enable remote readout of the temperature at any location on the device. Using a polydimethylsiloxane (PDMS)/glass chip, the temperature rise due to the exothermic reaction of hydrochloric acid and ammonia was locally monitored at the end of the microchannel where the reaction took place with a fiber probe. A temperature gradient imposed along a glass/glass chip was measured with spatial resolution down to ∼9 μm using confocal microscopy. Brites et al.^230^ also used Eu^3+^/Tb^3+^ codoped nanoparticles suspended in water to measure the temperature gradient imposed along a capillary tube with ∼65 μm spatial resolution (Figure 8b). Zhu et al.^231^ recently demonstrated 2D thermometry in microchannels using the temperature-dependent PL intensity of octylamine substituted CsPbBr_3_ perovskite QDs, including transient measurements of laminar water flow, with ∼10 μm spatial resolution and 200 ms temporal resolution. As a potential strategy for thermometry in microfluidic devices that would not require dispersing luminescent nanomaterials in the fluid, Savchuk et al.^232^ developed PDMS/NaYF_4_:Yb^3+^,Er^3+^ composites suitable for ratiometric thermometry that also maintain the transparency of PDMS.

Other nanofluid-based luminescence thermometry studies extend beyond microfluidic device applications. Brites et al.^14^ imposed temperature gradients along solutions of NaYF_4_:Yb^3+^,Er^3+^ UCNPs dispersed in water and chloroform and used ratiometric thermometry to record the time-dependent UCNP temperature rise at different positions, from which instantaneous Brownian velocities of the UCNPs were extracted. Luminescence thermometry has also been used to monitor the temperature rise of liquids heated by magnetic induction and by microwave irradiation, the latter of which has applications in areas including chemical synthesis, separations, and catalysis. Many magnetic induction heating studies have been driven by therapeutic hyperthermia applications, although additional motivating applications include catalysis and water purification.^233^ Shen et al.^234^ dispersed microwave-transparent thermochromic zinc-based MOFs in an ethanolic solution that was distributed in a 10 × 10 × 1 cm^3^ matrix consisting of 100 individual plastic cells (Figure 8c). When heated in a microwave cavity, temperature variations attributed to the nonuniform electric field were observed across the matrix.

Interestingly, a number of studies have reported local hotspots resulting from microwave or magnetic induction heating of nanofluids. These results are in contrast with heat transfer analysis demonstrating that the local temperature rise near electromagnetically heated nanoparticles is negligible, although the overall temperature rise for a macroscopic region can be appreciable due to the combined contributions of many individual nanoparticles.^235^ For example, Zhao et al.^236^ recently used microwave-absorbing carbon particles coated with Eu^3+^/Tb^3+^ mixed-metal organic complexes to measure the temperature rise in *n*-propanol and glycol-water solutions. The temperature in the *n*-propanol solution measured by ratiometric thermometry was 12 °C higher than that measured by a fiber-optic thermometer, which was taken as evidence of microwave-induced local hotspots. In another representative example, Dong and Zink^237^ encapsulated NaYF_4_:Yb^3+^,Er^3+^ UCNPs and superparamagnetic Fe_3_O_4_ nanocrystals together in mesoporous silica nanoparticles and used ratiometric thermometry to measure a temperature rise of 42 °C in an aqueous solution. Conversely, experiments using separate Fe_3_O_4_ nanocrystals and UCNPs in solution resulted in a measured temperature rise of only 19 °C, which was likewise considered indicative of local hotspots arising from magnetic induction heating. Similarly, Gu et al.^238^ recently reported an intracellular temperature rise of 8.0 °C measured by Sm^3+^/Eu^3+^ ratiometric luminescent thermometers chemically linked to magnetically heated Fe_2_O_3_ nanoparticles, whereas Sm^3+^/Eu^3+^ thermometers located near (but not chemically linked to) the Fe_2_O_3_ nanoparticles indicated an intracellular temperature rise of only 5.9 °C and the measured extracellular temperature was essentially unchanged. Further investigation is warranted to resolve this apparent contradiction between modeling and experimental results, as also highlighted recently by others.^239^

### 4.7 Other Applications

Finally, we briefly highlight a nonexhaustive selection of other emerging luminescence thermometry applications that do not fall fully within one of the categories covered previously and where the relevant literature may be less extensive. Given the micro to nanoscale spatial resolution of many luminescence thermometry techniques, fundamental studies of heat transfer at these length scales are a natural application. Although the existing work that will be discussed momentarily has largely focused on heat conduction scenarios in which Fourier’s law remains valid, experimental exploration of deviations from Fourier’s law are a logical next frontier.^240^ Savchuk et al.^241^ used time-resolved ratiometric thermometry measurements combined with a lumped capacitance model to determine the thermal resistance associated with heat dissipation in a laser-heated KLu(WO_4_)_2_:Ho^3+^,Tm^3+^ UCNP powder surrounded by air. Also using time-resolved ratiometric thermometry measurements, Rafiei Miandashti et al.^178^ studied the transient heating and cooling of gold-decorated NaYF_4_:Yb^3+^,Er^3+^ UCNPs on glass substrates surrounded by air or water and compared their results with the predictions of both analytical and finite element models.

Lin et al.^112^ developed nanocomposites consisting of Au nanoparticles that acted as heat sources and NaYbF_4_:Er^3+^/NaYF_4_ core/shell UCNPs and Ag_2_S QDs that served as thermometers with spectrally distinct temperature-dependent emission signatures. Surprisingly, a measured steady-state temperature difference of 30 °C across the ∼55 nm average distance separating the UCNPs and QDs was reported for nanocomposites in solution, and both the UCNPs and QDs measured temperatures tens of degrees higher than a thermocouple placed in the solution. These results are in contrast with analysis of similar scenarios showing that the local temperature rise of individual nanoparticles is negligible under continuous wave laser heating^235^ and that individual nanoparticles can be treated as thermally lumped to an excellent approximation.^53,176^ The times to reach a steady-state temperature as measured by the UCNPs, QDs, and thermocouple were also all on the order of tens to hundreds of seconds, at odds with the much faster time scale expected for heating of an individual nanoparticle but in line with the time scale expected for collective heating of the solution,^235^ suggesting that these measurements may call for closer examination.

Luminescence thermometry has also been applied to phase transitions such as the metal–insulator transition of VO_2_, where the question of whether the DC voltage- or current-induced phase transition is thermally or electronically driven has been strongly contested. To elucidate the role of Joule heating, Zimmers et al.^161^ used a single ∼1 μm Yb^3+^/Er^3+^ codoped fluoride glass particle to measure the local temperature rise, as noted in Section 3.3 (Single-Particle Measurements). The measured temperature consistently reached the phase transition temperature as the insulator to metal transition occurred, indicating that Joule heating plays a critical role. Brites et al.^242^ reported a geometric phase transition in water at ∼330 K that was identified using temperature-dependent instantaneous Brownian velocity measurements of NaYF_4_:Yb^3+^,Er^3+^ UCNPs.

Next-generation devices beyond those covered in prior sections may have different operating principles and, as a result, distinct metrology requirements. Errulat et al.^243^ demonstrated a Dy^3+^-based single-molecule magnet that also functioned as a luminescent thermometer under magnetic fields as high as 7 T, a potentially useful capability for temperature monitoring in future optomagnetic devices. Additional work in this growing area includes Dy^3+^-based single-molecule magnets that enable simultaneous luminescence thermometry and magnetic field sensing,^244^ as well as thermometry with enhanced temperature sensitivity achieved by combining temperature-dependent optical and magnetic signals,^245^ and Tb^3+^-based single-molecule magnets with luminescence thermometry capabilities.^246^ Thermal management and metrology of lithium-ion batteries is another ongoing challenge. Li et al.^247^ integrated an optical fiber sensor containing NaYF_4_:Yb^3+^,Er^3+^/NaYF_4_ core/shell UCNPs into a rechargeable lithium-ion battery and monitored the battery temperature in real time for different discharge rates. Luminescence thermometry has also been applied to inform thermal management strategies for optoelectronics and integrated photonics^248^ and guide the design of thermally actuated photonic devices;^249^ alternatively, optoelectronic devices themselves can also serve as thermometers.^250^ While UCNPs have longstanding anticounterfeiting applications based on their unique optical properties, Maturi et al.^251^ recently reported QR codes containing NaGdF_4_:Yb^3+^,Er^3+^ UCNPs that rely on emission changes resulting from laser-induced heating as part of a two-step verification process.

Although quantum coherence control typically requires low temperatures, Liu et al.^252^ demonstrated coherent control of the electron spins of NV centers in nanodiamonds at temperatures near ∼1000 K using pulsed laser heating. Microwave pulses much shorter than the nanodiamond thermal time constant were used to manipulate the NV center spin state immediately following the laser heating, such that the nanodiamond remained near its peak temperature. To estimate nanodiamond temperatures above 700 K, where fluorescence quenching prevents ODMR thermometry, ODMR measurements were performed after allowing the nanodiamond to partially cool. The peak temperature was subsequently calculated using an analytical transient heat transfer model. In contrast with studies of laser-induced heating, Roder et al.^253^ used ratiometric thermometry to assess the temperature decrease of optically trapped upconverting YLF:Yb^3+^,Er^3+^ nanocrystals in liquid media resulting from laser refrigeration. Due to particle-to-particle variation in the ratiometric thermometry signal and the inability to calibrate the trapped particle, quantitative ratiometric thermometry measurements were not feasible, but the luminescence intensity ratio was shown to increase as a function of 975 nm laser excitation intensity (indicative of heating) and decrease as a function of 1020 nm laser excitation intensity (indicative of cooling). These final two works exemplify demanding applications that have taken advantage of luminescence thermometry capabilities such as high-temperature and single-particle measurements, while simultaneously underscoring opportunities for continued materials and technique development to address challenging metrology needs.

## 5 Emerging Areas

### 5.1 Measurement Artifacts

The growth of luminescence thermometry applications has been accompanied by an increasing number of reports identifying various measurement artifacts that, without careful calibration and correction, can lead to errors in the measured temperature values often on the order of tens of degrees or more. Artifacts specific to biological applications have been reported, such as spectral distortions due to tissue absorption and scattering,^113,254^ and Bednarkiewicz et al.^255^ reviewed different sources of such artifacts and offered guidelines for avoiding or mitigating artifacts in a biological context. Luminescence thermometry artifacts relevant to both biological and nonbiological applications were also discussed in detail in a recent review by Brites et al.^4^ Below, we summarize research on luminescence thermometry measurement artifacts relevant to a broad array of applications, the majority of which pertains to the commonly used ratiometric thermometry signal of Yb^3+^/Er^3+^ codoped UCNPs.

One potential source of artifacts is the optical instrumentation used to perform the luminescence measurements, such as spectral distortions caused by high numerical aperture objective lenses^4^ or noise from the CCD sensor used to record spectra.^66,67^ The resulting temperature errors reported in these cases ranged from less than one degree up to tens of degrees. Optical misalignment can also introduce artifacts. For example, deviations on the order of several degrees were observed during temperature mapping via NaYF_4_:Yb^3+^,Er^3+^ UCNPs over scan distances of ∼100 μm.^256^

**Figure 9:**
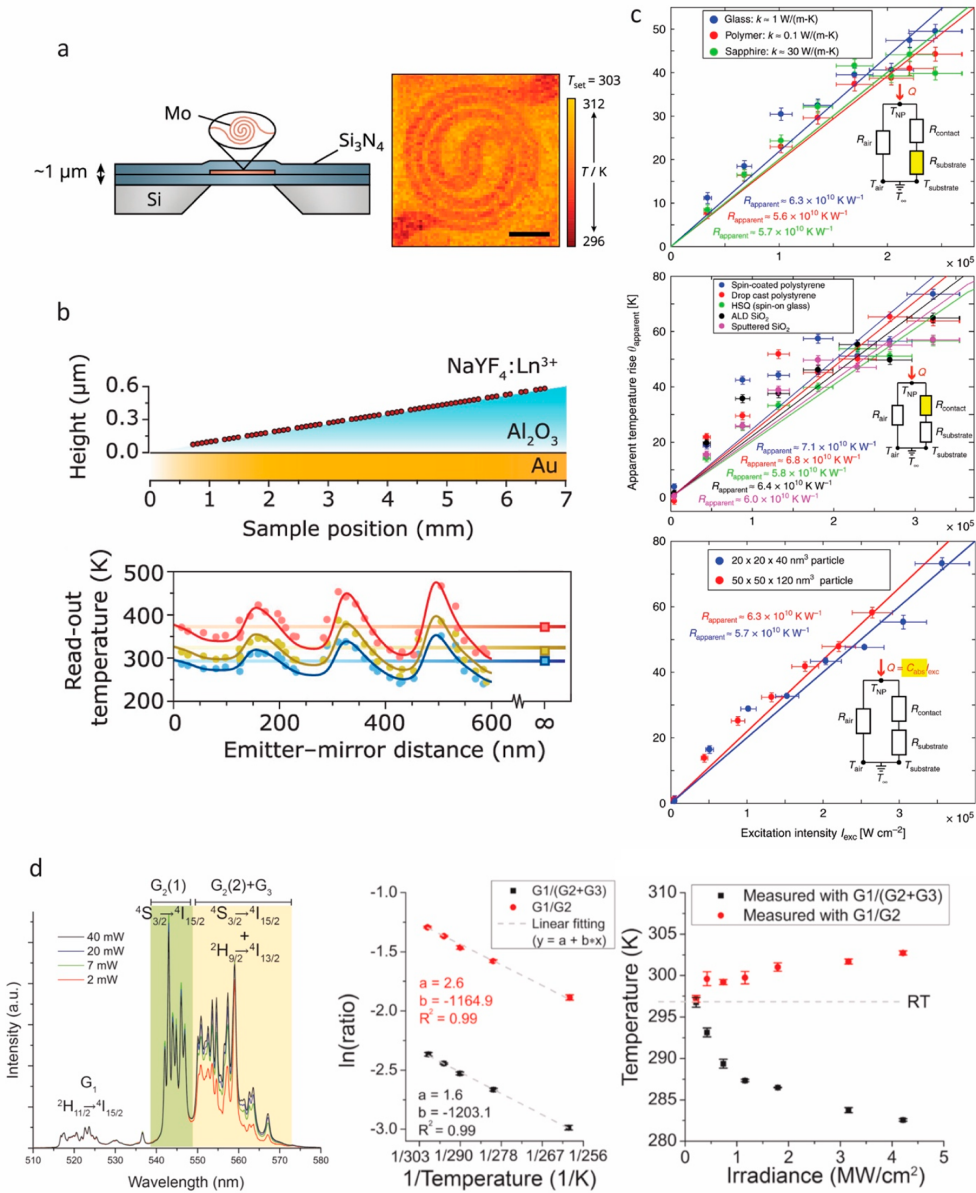
(a) Metallic surfaces such as the Mo spiral microheater shown here can act as a mirror, resulting in interference between the direct emission of the luminescent thermometers and the reflected emission. This phenomenon led the NaYF_4_:Yb^3+^,Er^3+^ UCNPs on the spiral microheater surface to erroneously report a lower temperature than those off the heater, an artifact that must be corrected to obtain accurate temperature measurements. Reprinted with permission from ref (54) Copyright 2021 American Chemical Society. (b) Fabricating samples consisting of UCNP monolayers located at controlled, variable distances from Au mirrors later allowed for systematic studies of the same interference phenomenon described in (a), which demonstrated how the measured temperature oscillates as a function of the emitter-mirror distance. Reprinted with permission from ref (55) Copyright 2023 American Chemical Society. (c) Apparent temperature rises exceeding 50 K were measured using individual NaYF_4_:Yb^3+^,Er^3+^ UCNPs at the high excitation intensities required for single-particle measurements. However, this apparent self-heating was unaffected by manipulating the UCNP heat dissipation pathways or absorption cross-section, indicating that this effect is a nonthermal artifact. Reprinted with permission under a Creative Commons CC BY 4.0 license from ref (53) Copyright 2018 Springer Nature. (d) The emission intensity from Yb^3+^/Er^3+^ codoped UCNPs remains constant with laser power in the first portion of the wavelength range corresponding to the ^4^S_3/2_ to ^4^I_15/2_ Er^3+^ transition but increases in the second portion due to undesirable contributions from the ^2^H_9/2_ to ^4^I_13/2_ transition. Consequently, calculations of the temperature-dependent ratio that include both portions of this wavelength band can lead to erroneous measurements of decreasing temperatures as a function of laser irradiance. Reprinted with permission from ref (260) Copyright 2021 Elsevier B.V. All rights reserved.

Artifacts can also originate from the photonic environment in which the nanoparticle thermometers are placed. Van Swieten et al.^54^ demonstrated temperature readout errors of more than 10 K from NaYF_4_:Yb^3+^,Er^3+^ UCNPs on a Mo spiral microheater embedded in a Si_3_N_4_ membrane due to reflectivity differences between Mo and Si_3_N_4_ (Figure 9a). The microheater surface acted as a mirror, leading to interference between the direct emission from the UCNPs and the reflected emission. Because the emission wavelengths for the two Er^3+^ transitions used for ratiometric thermometry vary slightly, this interference affects the emission from each transition differently, thereby altering the temperature-dependent ratio. Later, Vonk et al.^55^ fabricated samples consisting of UCNP monolayers located at controlled, variable distances from Au mirrors and showed that in this scenario, the same interference phenomenon could lead to temperature errors of up to ∼100 K for Yb^3+^/Er^3+^ codoped UCNPs and ∼250 K for Ho^3+^-doped UCNPs (Figure 9b). Photonic artifacts caused by scattering from purely dielectric polystyrene microspheres that resulted in temperature errors of ∼10 K were also demonstrated.

Various artifacts caused by raising the laser excitation intensity have been reported. Pickel et al.^53^ observed an increase in the ratiometric thermometry signal for individual NaYF_4_:Yb^3+^,Er^3+^ UCNPs that would indicate a temperature rise over 50 K if interpreted as thermal (Figure 9c). Directly manipulating the UCNP heat dissipation pathways and absorption cross-section had no effect on this apparent temperature rise, demonstrating that the effect is nonthermal. Complementary rate equation modeling instead showed that a combination of radiative and nonradiative relaxation from higher-lying Er^3+^ energy levels that become heavily populated at high single-particle excitation intensities increases the temperature-dependent ratio. Working in a lower excitation intensity range, Nguyen et al.^257^ instead identified a decrease in the ratio with excitation intensity that was attributed to decreased population of the ^2^H_11/2_ excited state. Several works have identified emission from the ^2^H_9/2_ to ^4^I_13/2_ transition of Er^3+^, which is frequently observed at higher excitation intensities and overlaps spectrally with the ^4^S_3/2_ to ^4^I_15/2_ Er^3+^ transition used for ratiometric thermometry, as a source of measurement artifacts.^162,258−260^ Because the ^2^H_9/2_ to ^4^I_13/2_ transition results from a three-photon upconversion process, the emission from this transition displays a different excitation intensity scaling than the Er^3+^ transitions used for ratiometric thermometry that originate from two-photon upconversion processes, leading to errors in temperature measurements where the excitation intensity is varied (Figure 9d).

The appropriate correction methodology depends on the source of the artifacts. Some excitation intensity-dependent artifacts can be avoided or mitigated by working at fixed or low excitation intensities. Another approach is to correct the ratio by subtracting or dividing out an excitation intensity-dependent term.^257,261^ Martins et al.^258^ also demonstrated the disappearance of the emission from the ^2^H_9/2_ to ^4^I_13/2_ transition when the excitation wavelength was switched from 980 to 808 nm. Alternatively, deconvolution^258^ or experimental procedures^54,162^ can be applied to separate and subsequently remove the emission originating from the ^2^H_9/2_ to ^4^I_13/2_ transition prior to calculating the ratio.

Beyond artifacts related to the ratiometric thermometry signal for Yb^3+^/Er^3+^codoped UCNPs, other artifacts relevant to this composition have also been identified. For example, Yb^3+^/Er^3+^codoped UCNP lifetimes have been shown to display nonthermal dependence on excitation intensity^53^ and proximity to a dielectric interface.^166^ Furthermore, in addition to artifacts and correction methods specific to Yb^3+^/Er^3+^ codoped UCNPs, artifacts related to other UCNP compositions and alternative strategies to avoid artifacts have been identified. Labrador-Páez et al.^262^ reported errors in temperature measurements based on IR emitting Tm^3+^-doped and Nd^3+^-doped luminescent thermometers caused by variations in parameters including the excitation intensity, local concentration of the thermometers, optical path length, and optical absorption of the surrounding medium. Another strategy to avoid artifacts related to wavelength-dependent spectral distortions is to perform ratiometric thermometry using two spectrally overlapping emission bands that can be separated along another axis. Qiu et al.^113^ reported a novel nanothermometer based on a hybrid structure composed of PbS QDs and NaYbF_4_:Tm^3+^ UCNPs both emitting near 810 nm. Because the PbS QDs have ns lifetimes while the NaYbF_4_:Tm^3+^ UCNPs have μs lifetimes, the two emission signals could be separated via time-gated spectroscopy.

### 5.2 Advanced Data Analysis and Machine Learning (ML) Approaches

In contrast with extracting temperature from a single temperature-dependent luminescence feature such as spectral peak intensity, peak width, lifetime, or the intensity ratio of two emission bands, advanced data analysis and ML techniques can combine multiple features to enhance temperature sensitivity and measurement accuracy. Approaches such as multiparametric sensing, multiple linear regression, dimensionality reduction, and neural networks (NNs) can transform original data into quantities that have no interpretable physical meaning, but have the potential to require fewer calibration temperatures and produce temperature readouts with smaller uncertainty than traditional calibration methods.

Choi et al.^88^ performed multiparametric analysis based on a linear combination of the peak position, width, and relative amplitude of the ZPL for nanodiamonds containing a high concentration of SiV centers. A noise floor of 13 mK/Hz^1/2^ was achieved, resulting in a readout speed orders of magnitude faster than other all-optical methods with similar spatial resolution and temperature uncertainty. Chen et al.^263^ used multiple linear regression to improve the temperature sensitivity and reduce the temperature uncertainty of Mn^2+^/Tb^3+^ codoped Ca_2_LaTaO_6_ luminescent thermometers. Maturi et al.^264^ applied multiparametric analysis and multiple linear regression to thermometry based on green fluorescent protein and Ag_2_S nanoparticles, demonstrating temperature uncertainties as low as 0.05 K. Ximendes et al.^265^ applied a dimensionality reduction approach to calibration data sets obtained from nanoparticles containing Yb^3+^, Nd^3+^, and Er^3+^ and Ag_2_S nanoparticles. For the rare earth-containing nanoparticles, two intensity ratios and one peak intensity were calibrated as a function of temperature and both linear and nonlinear transformations (principle component analysis and t-distributed stochastic neighbor embedding, respectively) were applied to the data. These dimensionality reduction approaches reduced the temperature uncertainty to 0.09 °C compared to 0.15 °C for a conventional approach.

Stone et al.^266^ synthesized nanodiamonds containing GeV and SiV centers and used the temperature dependence of the ZPL position and width for both types of colors centers as inputs to a ML multifeature regression model. Using only three to five temperature-dependent features and three calibration temperatures for a set of five nanodiamonds, their model was able to predict the temperature of any other nanodiamond with 1.5 K uncertainty and a noise floor of 1.1 K/Hz^1/2^. Liu et al.^267^ reported the application of NN recognition to extract the temperature from the magnitude and shape of Rhodamine B fluorescence spectra. Here, two different types of NNs were trained, one based on the integrated intensity and peak intensity, and the other based on the normalized spectra, which represented the spectral shape. The shape-based NN performed better than the intensity-based NN in measurement accuracy by a factor of 3 (noise floor of 4.2 mK/Hz^1/2^ vs 13 mK/Hz^1/2^). This improved performance resulted from eliminating the effect of fluorescence intensity fluctuations and using a greater number of temperature-dependent features to determine the temperature. Later, the same methodology and material were applied in a transient study to measure the temperature rise due to heating from a pulsed laser. A pump–probe approach was implemented to achieve 10 ns temporal resolution and the results were well-described by a 1D thermal diffusion model.^268^ Munro et al.^103^ further optimized NN inputs and found that NNs trained on a combination of peak intensity-based and shaped-based inputs offered better accuracy than peak intensity-based or shaped-based inputs alone. In addition to spectral features, lifetime decay curves can also be incorporated as inputs. Lewis et al.^100^ combined normalized spectra and lifetime decay curves of CdTe QDs in a microfluidic device. Different NNs were established, such as a conventional NN, a dense fully connected neural network (DNN) that was fed only spectra or lifetime decay curves, and a DNN that was fed both spectra and lifetime decay curves in separate branches. Compared to the other NNs, the DNN that was fed spectra and lifetime decay curves together yielded the lowest error. Applications of other new ML models such as convolutional NNs^269^ and automated ML tools^270^ to luminescence thermometry have also been reported, and further research in this burgeoning area will undoubtedly follow.

### 5.3 Batch Calibration and Particle-to-Particle Uniformity

As discussed in Section 5.1 (Measurement Artifacts), growing recognition of various ways in which the surrounding environment and excitation conditions can influence luminescence thermometry signals has led to the development of alternative calibration procedures to either account for or avoid these effects. A related challenge that has received less attention is errors or uncertainty resulting from applying the calibration curve obtained for a given particle or ensemble either to different particles from the same batch or another batch of nominally identical particles. Such issues are particularly relevant for single-particle measurements, where there is no averaging among particles and particle-to-particle heterogeneity is inherently important. However, these concerns are also relevant for ensemble measurements. The motivations for applying a given calibration curve to other particles from the same batch or to a new batch are both practical and fundamental: performing a separate calibration for every measurement can be tedious and decreases the appeal of the technique and its potential for broader deployment. Furthermore, in certain applications, prior calibration of same particles used for the final temperature measurement may be impractical or impossible. For example, in catalysis applications where the goal is to measure the local temperature rise during a chemical reaction, calibration of the exact particles used for these measurements is often impossible since raising the sample temperature could inadvertently catalyze the reaction, an irreversible process.^206^ Similarly, in optical trapping and levitation experiments, particles are trapped at random and thus cannot be calibrated beforehand.

**Figure 10:**
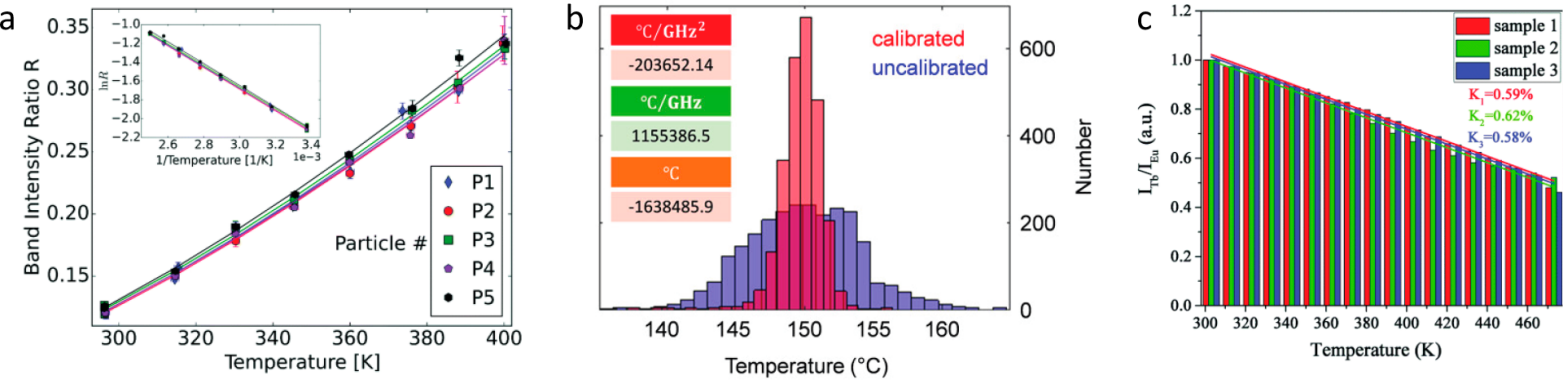
(a) Luminescence intensity ratio vs temperature calibration for five individual 20 × 20 × 40 nm^3^ NaYF_4_:Yb^3+^,Er^3+^ UCNPs. Calibrating multiple single particles allows for estimation of the temperature uncertainty associated with batch calibration, in which previously measured calibration curves for particles from a given batch are applied to an uncalibrated particle from the same batch. Reproduced from ref (17) with permission from the Royal Society of Chemistry. (b) Individually calibrating the temperature-dependent zero-field splitting for NV center-containing nanodiamonds used for ODMR thermometry reduces the temperature uncertainty compared with uncalibrated measurements. Reprinted with permission from ref (80) Copyright 2020 American Chemical Society. (c) Luminescence intensity ratio vs temperature calibrations for three different batches of Tb^3+^ and Eu^3+^ codoped MOFs, showing similar temperature-dependent responses across the three batches. Reproduced from ref (272) with permission from the Royal Society of Chemistry.

While many of the thermometry metrics described previously should in theory be uniform across different particles and batches of the same nominal composition, some variation is frequently observed in practice, even for probes like UCNPs with high particle-to-particle uniformity. Kilbane et al.^17^ calibrated the ratiometric thermometry signal for five individual NaYF_4_:Yb^3+^,Er^3+^ nanoparticles and evaluated both the smallest detectable temperature change for a single particle and the temperature uncertainty associated with applying these prior calibrations to another particle from the same batch (Figure 10a). The single-particle detection limits were estimated to be ±0.3 K at 296 K, ±1.4 K at 350 K, and ±3.3 K at 400 K., while the uncertainties associated with applying a batch calibration based on the five particles to another particle from the same batch were estimated to be ±1.2 K at 296 K, ±3.3 K at 350 K, and ±7.2 K at 400 K. Pickel et al.^53^ performed a similar analysis in which slopes obtained from fitting the measured ln(*r*) vs 1/*T* (a linear relationship) data for five individual NaYF_4_:Yb^3+^,Er^3+^ nanoparticles were used to evaluate uncertainties in the apparent temperature rise recorded for other single particles from the same batch. De Oliveira Lima et al.^157^ also performed ratiometric thermometry calibrations of two individual KGd_3_F_10_:Yb^3+^, Er^3+^ nanoparticles and calculated the relative sensitivity as a function of temperature for both particles.

Foy et al.^80^ used a wide-field technique that allows for simultaneous measurements of hundreds of NV center-containing nanodiamonds in parallel and evaluated the temperature-dependent zero-field splitting for 2573 nanodiamonds between 23.5 and 150 °C. The mean standard deviation of the thermal response across all temperatures was found to be 3.88 °C, greater than the 2.17 °C ± 0.46 °C uncertainty expected from measurement error, which is indicative of variations in the thermal responses of individual nanodiamonds. Applying individual calibrations to each nanodiamond improved the mean temperature uncertainty to 2.6 °C per ∼1 × 1 μm^2^ pixel (Figure 10b). As noted in Section 5.2 (Advanced Data Analysis and Machine Learning Approaches), Stone et al.^266^ demonstrated an alternative strategy based on a multifeature regression ML algorithm to facilitate all-optical thermometry using uncalibrated nanodiamonds codoped with GeV and SiV centers. Training the algorithm with as a few as five nanodiamonds enabled temperature measurements with 1.5 K uncertainty and a noise floor of 1.1 K/Hz^1/2^ for uncalibrated nanodiamonds. Although other approaches achieve lower temperature uncertainties and noise floors, this method offers a practical strategy for thermometry using uncalibrated nanodiamonds that minimizes the required number of prior calibration measurements. Bommidi and Pickel^85^ measured the excited state lifetimes of NV centers in five individual nanodiamonds each on silicon and glass substrates between 300 and 500 K. They evaluated nanodiamond-to-nanodiamond variation in the absolute lifetime values at a given temperature as well as in the change in lifetime over the temperature range investigated and the associated linearized temperature coefficients.

Other have evaluated variation among different batches of luminescent thermometers with the same nominal composition rather than within a single batch. Rao at el.^271^ demonstrated that three different batches of Tb^3+^ and Eu^3+^ codoped MOFs display similar temperature-dependent intensity ratios. Yang et al.^272^ reported comparable results for another Tb^3+^ and Eu^3+^ codoped MOF system (Figure 10c). Additional work has explored thermometry signals that are robust against changes in parameters like doping concentration, which may vary slightly across batches. Stefanska et al.^273^ showed that single-band ratiometric thermometry based on LaF_3_:Pr^3+^ powders is essentially independent of the Pr^3+^ doping for concentrations between 0.06% and 4.43%. Renero-Lecuna et al.^274^ evaluated the ratiometric thermometry signal based on emission from Stark sublevels in LaOCl:Nd^3+^ nanoparticles for Nd^3+^ concentrations between 1% and 10% and found that the slopes of the fitted ln(*r*) vs 1/*T* data were similar for all doping concentrations.

Although relatively few studies have considered particle-to-particle variation, it is clear that such variation frequently represents a non-negligible or even dominant source of uncertainty if temperature measurements are performed using particles that have not been previously calibrated. For single-particle studies, calibration measurements using multiple single particles should be reported. Similar strategies may also be applicable and relevant for ensemble measurements. For example, temperature calibrations can be performed at multiple spatial locations in the case of particles dispersed on a substrate or other solid sample. In temperature mapping applications, a pixel-by-pixel calibration may be required to achieve the highest possible accuracy. Emerging ML methods also represent an alternative strategy to overcome uncertainty and errors stemming from the use of uncalibrated luminescence thermometers while simultaneously avoiding practical challenges associated with performing numerous calibration measurements.

## 6 Summary and Outlook

While luminescence thermometry has both longstanding ties to and continued promise for biological applications, this applications-driven review has highlighted an extensive body work from the last several decades that demonstrates how luminescence thermometry can address challenges in many other areas. Identifying micro to nanoscale hotspots in electronics, an early device-oriented application of luminescence thermometry, remains a frontier opportunity, with increasingly important technologies such as wide bandgap devices presenting a new set of thermal challenges and metrology needs.^11,275^ Devices for quantum computing applications, many of which operate well below room temperature, similarly benefit from thermal characterization^276^ and are a prime example of applications that could take advantage of the wide operating temperature range of luminescent thermometers. Various applications in catalysis have appeared in recent years, indicating opportunities to optimize reactor conditions, delineate photothermal and photochemical mechanisms, and characterize the temperature rise resulting from heat generated by exothermic reactions.

Emerging areas of focus point to both opportunities and challenges for the field of luminescence thermometry. ML and other advanced data analysis methods suggest a path toward luminescence thermometry approaches that offer improved sensitivity and reliability while simultaneously requiring fewer calibration tasks. Increasing recognition of artifacts that can distort measured temperature values calls for carefully considered strategies to both identify and mitigate the effects of these artifacts, areas that are being addressed by a growing number of publications. While the presence of artifacts naturally raises questions about the robustness of luminescence thermometry and its ultimate potential for deployment in industrial or commercial settings, well-established and widely used techniques like resistance thermometry must also contend with confounding factors such as self-heating and strain-induced resistance changes, and strategies have been established to compensate and account for these effects.^277^ We have also emphasized challenges related to particle-to-particle uniformity and the resulting implications for batch calibration methods as a less-examined area that merits further consideration with an eye toward practical applications.

Luminescence thermometry frequently benefits from innovations in probe materials, and parallel developments in this area suggest intriguing possibilities. For example, recent advances in highly doped UCNPs^278^ have led to a variety of applications including bright probes for single-molecule studies,^279^ multiple super-resolution imaging modalities,^280−283^ and optical multiplexing schemes.^284^ Future exploration of these probes for thermometry applications may likewise yield exciting new capabilities. Avalanching nanoparticles^280,281^ offer particularly intriguing possibilities for thermometry since avalanche phenomena are highly sensitive to environmental perturbations like temperature changes, and simulations exploring the temperature-dependent processes involved in photon avalanching have yielded promising results.^285^ The confluence of advances in probe materials, instrumentation, and data analysis, combined with ongoing thermal characterization needs stemming from both established and early stage application categories, should provide a diverse set of impactful research directions for luminescence thermometry researchers to pursue in the years ahead.
